# Integrated transcriptomics and metabolomics reveal the effects of chronic alcohol consumption on periodontitis in rats

**DOI:** 10.3389/fimmu.2026.1735355

**Published:** 2026-05-18

**Authors:** Zirui Zhao, Zixuan Zhao, Yaqian Zhai, Jingyuan Sun, Na Liu, Qing Liu

**Affiliations:** 1 Hebei Key Laboratory of Stomatology/Hebei Technology Innovation Center of Oral Health, School and Hospital of Stomatology, Hebei Medical University, Shijiazhuang, Hebei, China; 2 Department of Preventive Dentistry, School and Hospital of Stomatology, Hebei Medical University, Shijiazhuang, Hebei, China

**Keywords:** chronic alcohol consumption, metabolomics, micro-CT, periodontitis, transcriptomics

## Abstract

**Introduction:**

Chronic alcohol consumption exacerbates the progression of periodontitis, the sixth most prevalent global disease affecting over 740 million people, though its underlying mechanisms remain poorly understood.

**Objective:**

The study aims to elucidate the impact and mechanisms of chronic alcohol consumption on periodontitis in rats through integrated transcriptomic and metabolomic analyses.

**Methods:**

Thirty-two male Wistar rats were randomly divided into four groups: control (Ctrl), periodontitis (Perio), alcohol (Alc), and alcohol with periodontitis (Perio+Alc). Chronic alcohol consumption and periodontitis models were established. Periodontal tissue structure was evaluated using hematoxylin-eosin (HE) staining and Micro-CT analysis. Transcriptome sequencing and untargeted metabolomics were employed to analyze transcriptomic and metabolic profiles in gingival tissues.

**Results:**

Chronic alcohol consumption significantly aggravated inflammatory infiltration and alveolar bone resorption in periodontal tissues. RNA sequencing identified 2,960 differentially expressed genes (DEGs) in the Perio+Alc and Perio groups. Metabolomics analysis revealed 611 metabolites in negative ion mode and 812 in positive ion mode. Integrative analysis demonstrated that alcohol exposure primarily influenced periodontal tissues through disruptions in four key pathways: beta-alanine metabolism, Arachidonic acid metabolism, Tryptophan metabolism and Aldosterone synthesis and secretion.

**Conclusion:**

Chronic alcohol consumption exacerbates periodontitis progression. This research elucidates transcriptomic and metabolomic alteration mechanisms, laying the theoretical groundwork for identifying therapeutic targets to mitigate alcohol-aggravated periodontitis.

## 1 Introduction

Periodontitis constitutes a major global public health challenge, ranking as the sixth most prevalent disease worldwide and affecting the oral health of over 740 million individuals (1). Between 1990 and 2019, the global age-standardized prevalence of severe periodontitis increased by 8.44% (2). This infectious and inflammatory disease, primarily affecting the periodontal tissues and initiated mainly by periodontal dysbiosis, is characterized by inflammation and progressive destruction of the tooth-supporting structures (3). It is further associated with various local and systemic risk factors and comorbidities (4).

Alcohol consumption poses a significant global health challenge, substantially contributing to the global burden of disease and imposing considerable healthcare and economic costs (5). Its intake is associated with numerous adverse health outcomes, including cardiovascular disease (6), nutritional deficiencies (7), cancer (8), accelerated aging (9, 10), and oral diseases (11, 12). Clinical evidence consistently demonstrates that alcohol consumption correlates with increased severity of clinical attachment loss (CAL) in a dose-dependent manner (13–15), and exhibits a positive association with more severe attachment loss and gingival bleeding (16). The detrimental impact of alcohol appears most pronounced on the gingiva, followed by the periodontal ligament and alveolar bone (13). Alcohol impairs neutrophil function (17), compromises macrophage and T-cell activity (18), thereby dysregulating the immune system, increasing susceptibility to severe infections, and disrupting bone homeostasis. Consequently, it accelerates alveolar bone resorption and modulates inflammatory response mechanisms in periodontitis (19). Current research indicates that chronic alcohol consumption adversely affects bone mineral density, calcium and phosphorus levels (20), and inhibits bone repair processes (21). However, the precise mechanisms underlying the influence of chronic alcohol consumption on periodontitis progression remain incompletely understood.

The integration of transcriptomic and metabolomic data provides a powerful approach for investigating complex disease mechanisms. In periodontitis research, transcriptomics and metabolomics have demonstrated considerable potential for identifying key genes and metabolites associated with the disease (22, 23), thereby enhancing understanding and enabling targeted interventions. Nevertheless, research specifically investigating the combined effects of chronic alcohol consumption and periodontitis remains relatively scarce. This study aims to systematically investigate the impact of chronic alcohol consumption on rat periodontitis and elucidate its underlying mechanisms using integrated transcriptomic and metabolomic approaches. The findings are expected to provide novel insights into the interplay between alcohol consumption and periodontitis and establish a foundation for developing new intervention strategies targeting alcohol-associated periodontitis.

## 2 Methods

### 2.1 Animal husbandry, grouping, and model establishment

**Figure 1:**
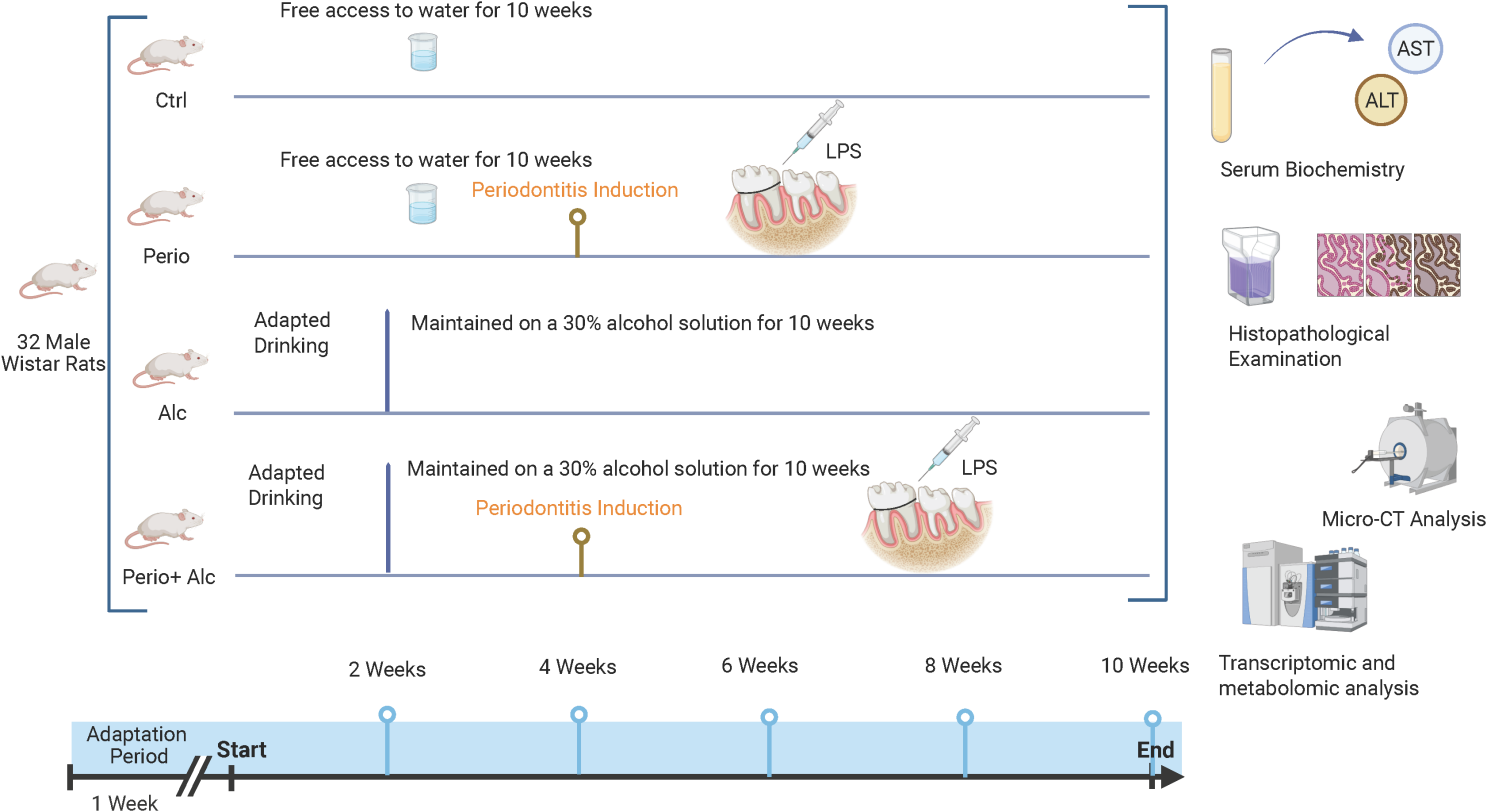
Schematic diagram of the experiment. After one week of acclimatization, rats were randomly assigned to 4 groups (n=8 per group): Control (Ctrl), Periodontitis (Perio), Alcohol (Alc), and Alcohol with Periodontitis (Perio+Alc). Rats in the Alc and Perio+Alc groups underwent gradual alcohol solution adaptation over 14 days (from 10% to 25%, v/v), followed by ad libitum access to 30%(v/v) alcohol solution for the subsequent 10-week experimental period, whereas the Ctrl and Perio groups received free access to water. Periodontitis was induced in the Perio and Perio+Alc groups at the end of week 4 via ligature wire combined with weekly local LPS injections for three weeks. All rats were sacrificed at week 10 for endpoint analyses, including serum biochemistry (ALT, AST), micro-CT assessment of alveolar bone loss, histopathology (HE staining), and tissue sampling for transcriptomics and metabolomics. This diagram was created with BioRender.com.

The experimental timeline and group allocation are summarized in **Figure 1**. 32 male SPF Wistar rats, aged 7–8 weeks old and weighing 200–220 g each, were obtained from Beijing Hfk Bioscience Co., Ltd. All rats were housed under controlled conditions (12-hour light/dark cycle, 20–23 °C) with ad libitum access to distilled water and standard chow. After 1 week of acclimatization, the rats were randomly assigned to four groups (n=8): control (Ctrl), periodontitis(Perio), alcohol (Alc), and alcohol with periodontitis (Perio+Alc). The Ctrl group received ad libitum water for 10 weeks. The Perio group received ad libitum water for 10 weeks and underwent periodontitis induction at the end of week 4. The Alc group received ad libitum 30% (v/v) alcohol solution for 10 weeks. The Perio+Alc group received ad libitum 30% (v/v) alcohol solution for 10 weeks and underwent periodontitis induction 4 weeks after initiating the 30% (v/v) alcohol-solution regimen. The study was approved by the Medical Ethics Committee of the Hospital of Stomatology Hebei Medical University.

The Alc and Perio+Alc group were gradually adapted to increasing concentrations of ethanol (v/v) over one week: 3 days with 10% (v/v) ethanol solution, followed by 3 days with 15% (v/v) ethanol solution, then 4 days with 20% (v/v) ethanol solution, and finally 4 days with 25% (v/v) ethanol solution. Thereafter, the rats had *ad libitum* access to 30% (v/v) ethanol throughout the 10-week study period.

Periodontitis was induced in the Perio group and the Perio+Alc group via a combination of ligature wire and local lipopolysaccharide (LPS) injection. Briefly, cotton thread and orthodontic ligatures were placed around the bilateral maxillary first molars. Using a microsyringe, 10 µL of LPS solution (1 mg/mL in sterile saline) was injected into both the buccal and palatal periodontal pockets of each ligated molar (two sites per tooth). Injections were performed once weekly for three consecutive weeks, resulting in a total LPS volume of 80 µL per rat per injection session.

### 2.2 Specimen collection and processing

At the end of the 10-week experimental period, rats were assessed for general health and periodontal tissue status. Following a 12-hour fast, blood samples were collected from the orbital venous plexus. Serum was isolated by centrifugation (10,000 r/min, 10 min, 4 °C) and stored at -80 °C for subsequent analysis. Rats were then humanely euthanized via intraperitoneal injection of an overdose of 0.6% sodium pentobarbital.

Periodontal tissues surrounding the right maxillary first molar were dissected, placed in cryovials, flash-frozen in liquid nitrogen for 5 minutes, and stored at -80 °C for transcriptomic and metabolomic analyses. The right maxillary alveolar bone was fixed in 4% paraformaldehyde (PFA) solution for micro-computed tomography (micro-CT) analysis. The left maxillary alveolar bone was fixed in 4% PFA solution for histopathological examination.

### 2.3 Biochemical analysis

Serum levels of alanine aminotransferase (ALT) and aspartate aminotransferase (AST) were measured using an automated biochemical analyzer (Mindray, Shenzhen, China) to evaluate liver abnormalities associated with chronic alcohol consumption.

### 2.4 Hematoxylin-eosin staining

Left maxillary alveolar bone specimens, fixed in 4% PFA at room temperature for 48 hours, were decalcified in 10% ethylenediaminetetraacetic acid disodium salt (EDTA) solution for 6 weeks. Following decalcification, tissues were embedded in paraffin, sectioned, and stained with hematoxylin and eosin (HE). Stained sections were examined under light microscopy for histological assessment.

### 2.5 Micro-CT scanning analysist

Micro-CT was used to perform layer-by-layer scanning of the right maxillary alveolar bone in each rat group. The acquired two-dimensional images were reconstructed into three-dimensional models using NRecon software. Subsequently, CT Analyser software was employed to measure the cementoenamel junction (CEJ) to alveolar bone crest (ABC) distances at six sites: mesial, central, and distal on both the buccal and palatal aspects of the right maxillary first molar. The mean CEJ-ABC distance was calculated.

The rectangular region of interest (ROI) was defined with the following dimensions: (1) height: 1 mm perpendicular to the CEJ boundary at the root surface, starting from the enamel-dentin junction; (2) length: 3.5 mm extending distally from the mesial boundary of the first molar’s enamel-dentin junction; (3) width: 2.5 mm along the bucco-palatal axis, with the root portion excluded using the software. Within this ROI, parameters including bone mineral density (BMD), bone volume fraction (BV/TV), trabecular number (Tb. N), and trabecular separation (Tb. Sp) were measured.

### 2.6 RNA extraction and gingival transcriptome analysis

Total RNA was extracted from rat gingival tissue samples (n=4) using TRIzol™ reagent (Invitrogen, 15596-026; Thermo Fisher Scientific, Shanghai, China). RNA integrity and concentration were assessed using the Agilent 2100 Bioanalyzer system (Agilent Technologies, California, USA). Subsequent reverse transcription and library construction were performed by Novogene (Beijing, China) following the manufacturer’s protocols (Illumina, San Diego, CA, USA). Gene expression levels were quantified as fragments per kilobase of exon per million mapped fragments using the StringTie software. Differential gene expression analysis identified significantly altered genes based on commonality and statistical significance, with differentially expressed genes (DEGs) defined by |log2FoldChange| ≥ 1 and P-adjust < 0.05 using DESeq2. Functional annotation and pathway enrichment analysis of DEGs were performed using Kyoto Encyclopedia of Genes and Genomes (KEGG) and Gene Ontology (GO) databases. For enrichment analyses, only terms containing at least 3 genes were retained, and a significance threshold of P < 0.05, generatio>0.05 was applied. All these filtering criteria were clearly defined to ensure the biological relevance and statistical reliability of the results.

### 2.7 LC-MS metabolomic analysis of gingival samples

Gingival tissue samples were sent to Novogene (Beijing, China) for Ultra-High Performance Liquid Chromatography-Mass Spectrometry (UHPLC-MS) analysis. Detailed methods for metabolite extraction, chromatographic separation, mass spectrometric analysis, and data processing are provided in the Methods section of **Supplementary Table 1**.

### 2.8 Integrated analysis of transcriptomic and metabolomic pathways

To identify coregulated biological pathways, we performed an integrated analysis by comparing the KEGG enrichment results from the independent transcriptomic and metabolomic datasets. Pathways significantly enriched in both analyses were defined as co-enriched pathways, representing the key biochemical and signaling pathways jointly perturbed at the gene and metabolite levels.

### 2.9 Statistical analysis of general data

All quantitative data were analyzed using SPSS 26. Results are presented as mean ± standard deviation (X± S). The normality of data distribution was assessed using the Shapiro-Wilk test, and the homogeneity of variances was verified using Levene’s test. For comparisons among the four groups, one-way analysis of variance (ANOVA) was applied when these assumptions were met. If a significant overall difference was indicated by ANOVA (p < 0.05), *post hoc* pairwise comparisons were performed using Tukey’s honestly significant difference (HSD) test. A p-value less than 0.05 was considered statistically significant.

## 3 Results

### 3.1 Chronic alcohol consumption induces hepatic injury and exacerbates periodontal pathology

**Figure 2:**
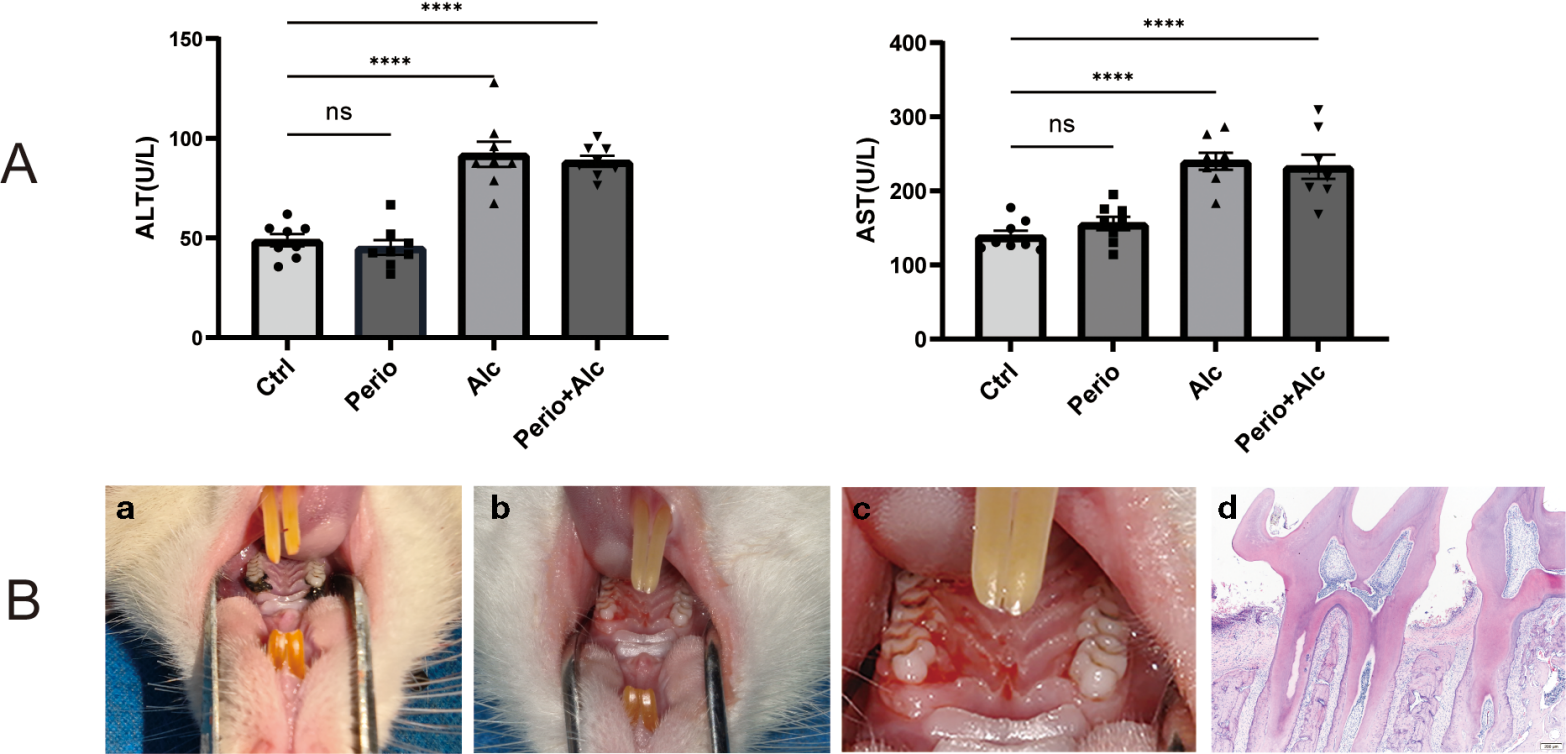
Features of alcohol-induced hepatic injury and periodontal pathology in rat models. **(A)** Serum levels of alanine aminotransferase (ALT) and aspartate aminotransferase (AST) across experimental groups. Data (mean ± SD) show a significant increase (****P < 0.0001) in the Alc and Perio+Alc groups compared to the Ctrl group. **(B)** Characterization of the periodontitis model. **(a–c)** Clinical manifestations showing inflamed and swollen gingiva. **(d)** Histopathology (HE, 40×) reveals classic periodontal breakdown, including apical migration of the junctional epithelium, inflammatory infiltrate, and alveolar bone resorption. ns, not significant.

Six weeks after ligature placement, the condition of the rat periodontal tissues was assessed. Significant dental calculus deposition, pigmentation, and food debris were visible around the cervical region of the bilateral maxillary first molars. The gingival tissue appeared dark red and swollen, with bleeding upon probing. Periodontal pockets were detectable, and varying levels of tooth loosening were observed. Histopathological examination (HE staining) revealed periodontal pocket formation, apical proliferation of the junctional epithelium, extensive inflammatory cell infiltration within the periodontal tissues, and reduced alveolar bone crest height. These findings collectively indicate the successful establishment of the periodontitis model (**Figure 2**).

Serum ALT and AST levels were significantly elevated in the Alc group and Perio+Alc group compared to the Ctrl group (*P* < 0.01), indicating alcohol-induced hepatic impairment. In contrast, no significant differences in these marker enzymes were observed between the Perio group and the Ctrl group (*P* > 0.05) (**Figure 1**).

### 3.2 Chronic alcohol consumption exacerbates periodontal inflammation in rats with periodontitis

**Figure 3:**
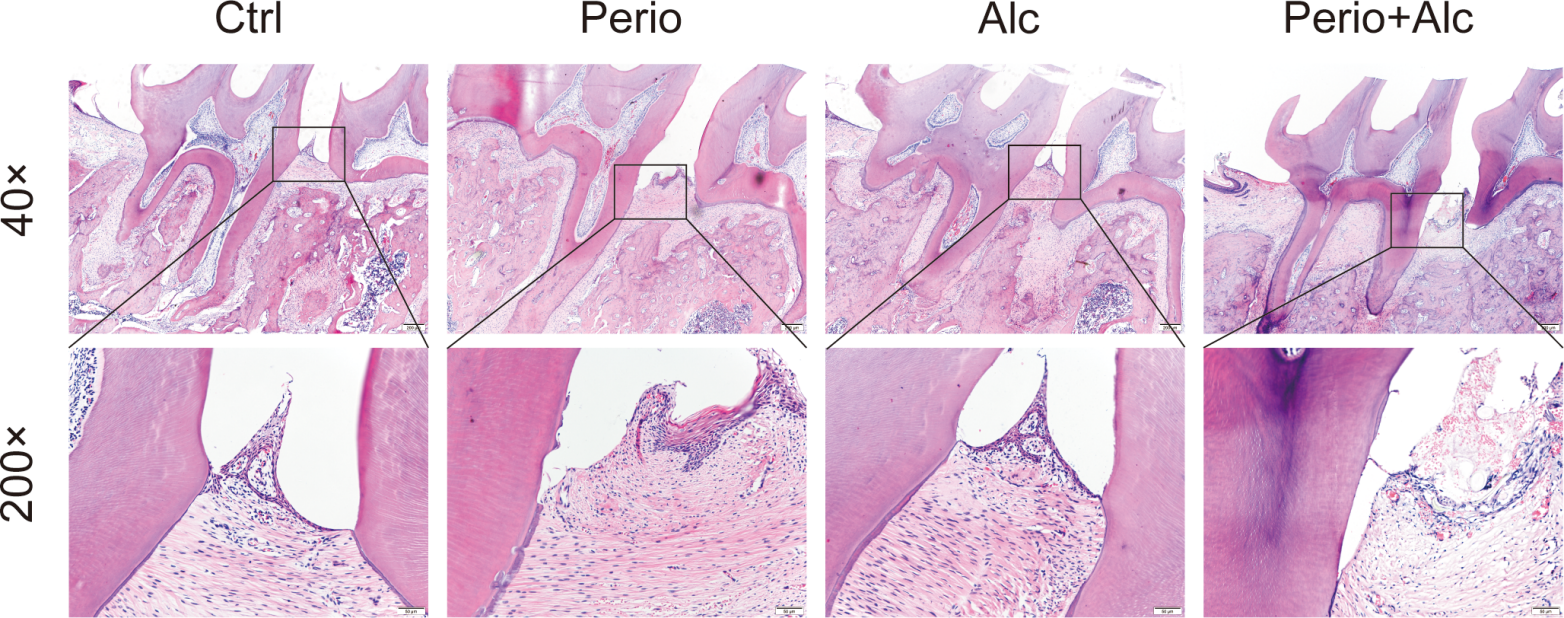
Periodontal tissue morphology across experimental groups (HE staining, 40×, 200×). The Ctrl group displays intact gingival epithelium, orderly periodontal ligament fibers, and normal alveolar bone. The Perio group exhibits classic periodontitis pathology, including apical migration of the junctional epithelium, inflammatory infiltration, and alveolar bone resorption (increased CEJ-ABC distance). The Alc group shows largely preserved tissue integrity with minimal inflammation. The Perio+Alc group presents the most severe destruction, characterized by extensive epithelial migration, dense inflammatory infiltrates, and the greatest degree of alveolar bone loss.

HE staining analysis reveals that the Ctrl group displayed intact gingival epithelium, with the junctional epithelium located at the cementoenamel junction. Periodontal ligament and collagen fibers were orderly arranged, with minimal inflammatory cell infiltration. The alveolar bone structure remained intact, showing no signs of resorption. In contrast, the Perio group exhibited apical migration of the junctional epithelium, resulting in attachment loss. This was accompanied by inflammatory cell infiltration within both the epithelium and lamina propria, edema and degeneration of periodontal ligament and collagen fibers, an increased cementoenamel junction to alveolar bone crest (CEJ-ABC) distance, and evident alveolar bone resorption. The Alc group demonstrated predominantly intact gingival epithelium with minimal inflammatory cell infiltration and no significant change in the CEJ-ABC distance. The most severe periodontal destruction was observed in the Perio+Alc group. This group exhibited attachment loss, apical migration of the junctional epithelium, extensive inflammatory cell infiltration throughout the epithelium and lamina propria, edema and disorganization of periodontal ligament and collagen fibers, the greatest CEJ-ABC distance, and the most pronounced alveolar bone resorption. Collectively, these findings indicate that chronic alcohol consumption induces significant destruction of periodontal tissues and promotes alveolar bone resorption (**Figure 3**).

### 3.3 Chronic alcohol consumption aggravates alveolar bone loss in rats with periodontitis

Micro-CT analysis revealed significant differences in alveolar bone resorption of the maxillary first molars among the groups. Compared to the Ctrl group, the Perio group exhibited a significantly increased bone resorption (*P* < 0.0001). In contrast, bone resorption in the Alc group did not differ significantly from the Ctrl group (*P* > 0.05). Notably, the Perio+Alc group demonstrated the most severe alveolar bone loss, characterized by the most pronounced root bifurcation exposure and a significantly increased CEJ-ABC distance compared to the Ctrl group (P < 0.0001). Furthermore, bone resorption in the Perio+Alc group was significantly higher than in the Perio group (*P* < 0.05).

BMD was significantly reduced in both the Perio and Alc groups relative to the Ctrl group(*P* < 0.05). The Perio+Alc group exhibited the most pronounced decrease in BMD, significantly lower than both the Ctrl group (P < 0.0001) and the Perio group (P < 0.05).

Compared to the Ctrl group, the Perio group exhibited significant decreases in BV/TV and Tb.N (*P* < 0.05). Compared to the Ctrl group, these parameters were further decreased in the Perio+Alc group (*P* < 0.001). Although the Alc group showed reductions in BV/TV and Tb.N relative to the Ctrl group, these changes were not statistically significant (*P* > 0.05). Similarly, despite lower BV/TV and Tb.N values in the Perio+Alc group compared to the Perio group, no significant difference was observed (*P* > 0.05).

**Figure 4:**
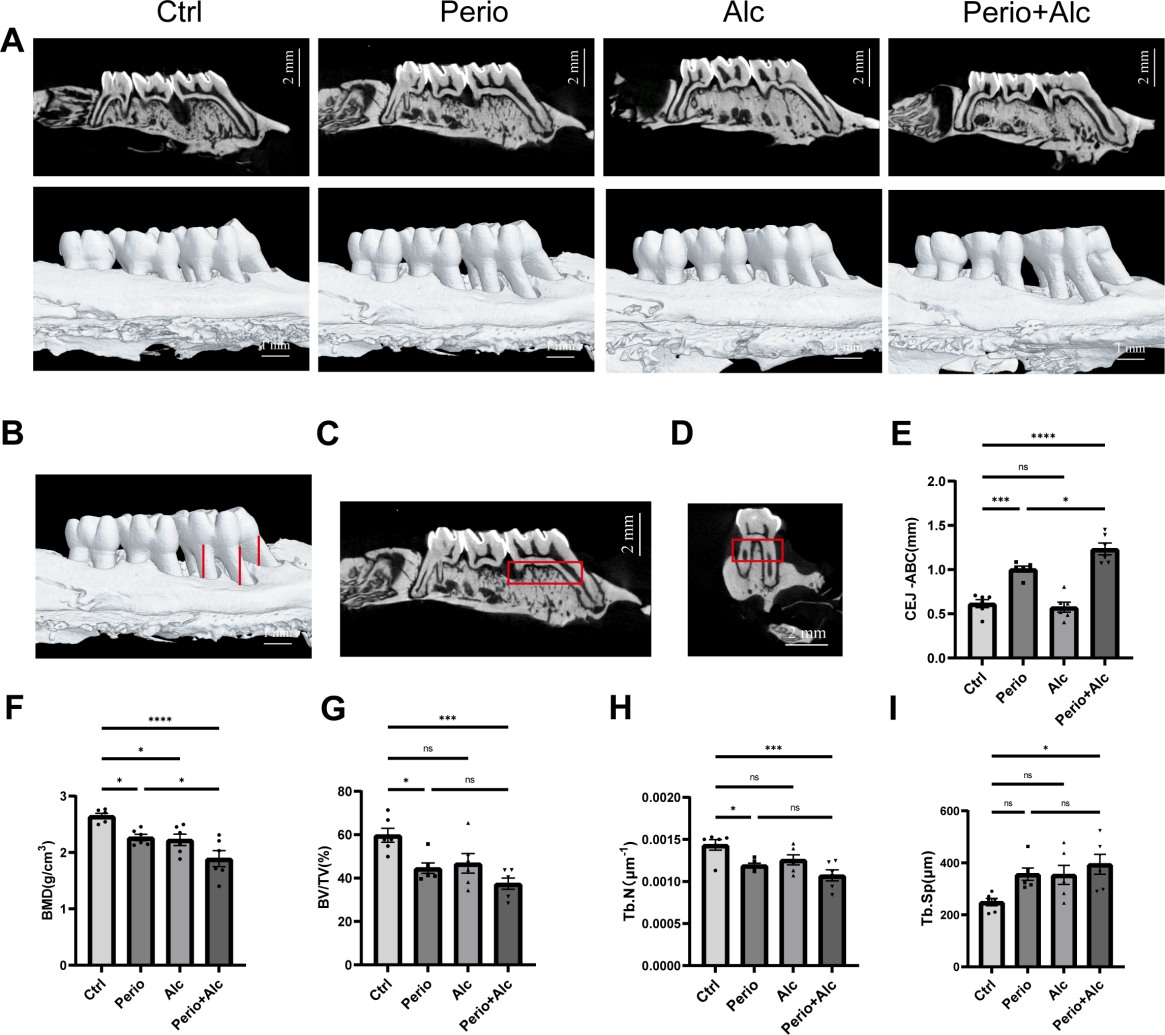
Micro-CT imaging and morphometric analysis of the maxillary alveolar bone. **(A)** Micro-CT 3D reconstruction image. **(B)** Measurement site of cementoenamel junction (CEJ) to alveolar bone crest (ABC) distances distance. **(C, D)** Coronal and transverse views defining the volumetric region of interest (ROI). **(E)** CEJ-ABC distance in all experimental groups. **(F)** Bone mineral density (BMD). **(G)** Bone volume fraction (BV/TV). **(H)** Trabecular number (Tb. N). **(I)** Trabecular separation (Tb. Sp) (*P<0.05; **P<0.01; **P<0.001; ****P<0.0001, ANOVA followed by Tukey *post hoc* test).

Compared with the Ctrl group, both the Perio and Alc groups showed increased Tb.Sp, although neither group exhibited a statistically significant increase (*P* > 0.05). The Perio+Alc group exhibited the most pronounced increase in Tb.Sp, which was significantly higher than that in the Ctrl group (*P* < 0.05). However, the difference in Tb.Sp between the Perio+Alc and Perio groups did not reach statistical significance (*P* > 0.05) (**Figure 4**).

### 3.4 Chronic alcohol consumption impairs redox homeostasis and energy metabolism in periodontitis

**Figure 5:**
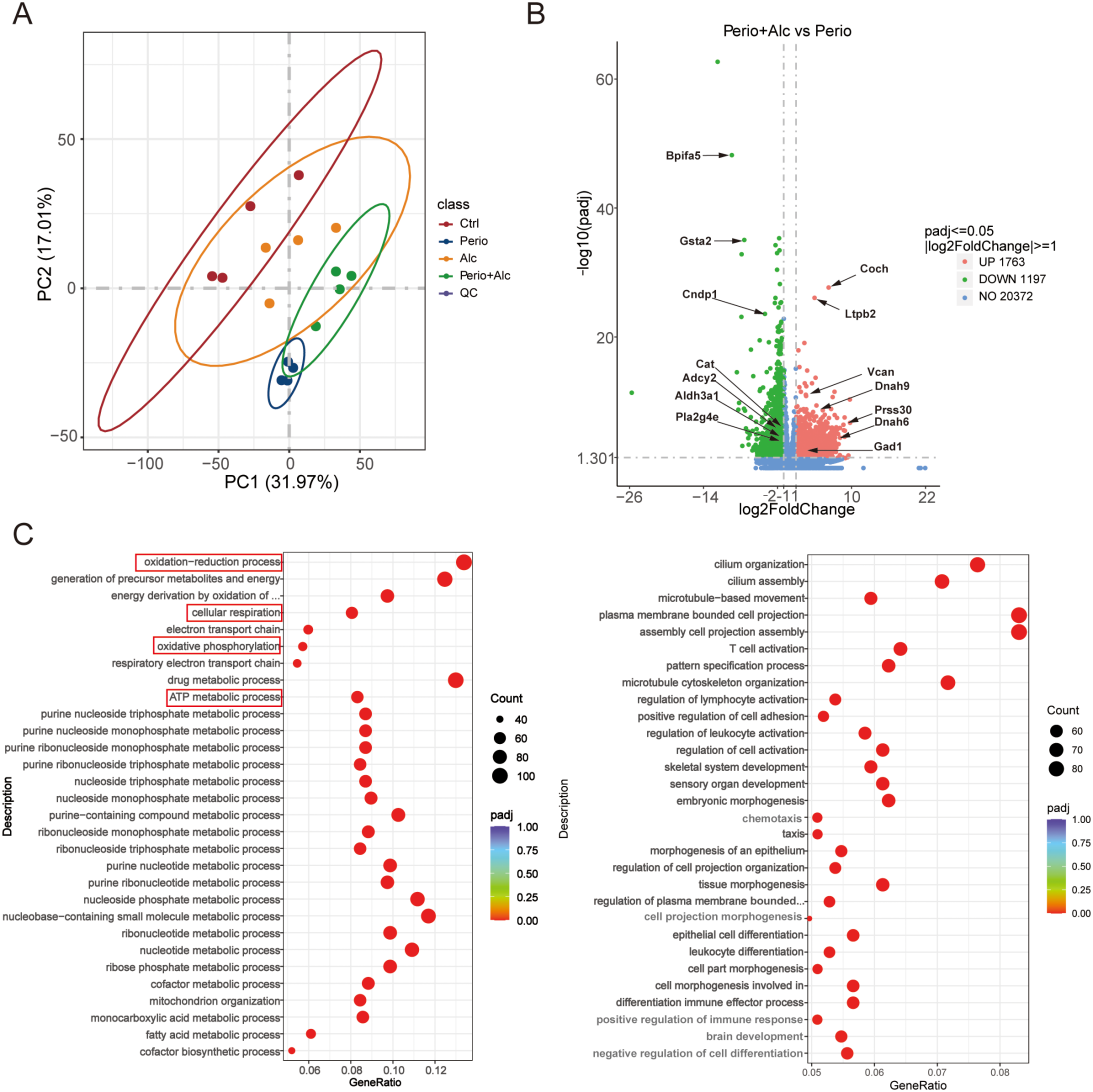
Effects of alcohol consumption and periodontitis on the gingival transcriptome. **(A)** Principal component analysis (PCA) plot of all RNA-seq samples, demonstrating distinct clustering and clear separation among the four experimental groups based on global gene expression profiles. **(B)** The volcano plot exhibits the most extensive transcriptional alteration in the Perio+Alc vs. Perio comparison, with 2,960 DEGs (1,763 upregulated, 1,197 downregulated). In volcano plot, red and blue dots denote significantly upregulated and downregulated genes, respectively (threshold: |fold change (FC)| ≥ 2 and P-adjust < 0.05). **(C)** Gene Ontology (GO) enrichment analysis of downregulated (left) and upregulated (right) differentially expressed genes in the Perio +Alc vs. Perio comparison. In each plot, the x-axis represents the Gene Ratio (the number of differentially expressed genes annotated to a specific GO term divided by the total number of differentially expressed genes). The y-axis lists the enriched GO biological process terms. The size of each bubble corresponds to the number of annotated genes, and the color gradient (from red to purple) represents the enrichment significance (adjusted P value), with red indicating the highest significance (P < 0.05, GeneRatio > 0.05, |FC|> 2).

RNA sequencing of gingival tissue samples across the four experimental groups identified 25,014 expressed genes. Principal component analysis (PCA) revealed distinct clustering of the samples based on alcohol intake and periodontal disease status, effectively segregating the four groups (**Figure 5A**). Differential expression analysis identified 1,922 DEGs between the Perio group and the Ctrl group, comprising 888 upregulated and 1,034 downregulated genes (**Supplementary Figure S1A**). The Alc group exhibited only 39 DEGs relative to the Ctrl group, of which 33 were upregulated and 6 were downregulated (**Supplementary Figure S1B**). Furthermore, comparison of the Perio+Alc group with the Perio group revealed a significantly larger set of 2,960 DEGs, including 1,763 upregulated and 1,197 downregulated genes (**Figure 5B**).

To functionally characterize the significant DEGs induced by chronic alcohol consumption and periodontitis, GO enrichment analysis was conducted. This analysis revealed distinct biological processes associated with each comparison group. Specifically, DEGs between the Ctrl and Perio groups were predominantly associated with glycosylation biological processes (**Supplementary Figure S1C**). In contrast, DEGs between the Ctrl and Alc groups were primarily enriched in biological processes related to spindle assembly, microtubule cytoskeleton organization, and mitotic cell cycle process. (**Supplementary Figure S1D**). Furthermore, DEGs between the Perio and Perio+Alc groups were centered on biological processes involving oxidation-reduction process, cellular respiration, oxidative phosphorylation, and ATP metabolic process biological processes (**Figure 5C**).

To elucidate the biological pathways associated with the identified genes, KEGG pathway enrichment analysis was conducted. This analysis revealed that differentially expressed genes (DEGs) between the Ctrl and Perio groups were predominantly enriched in protein processing in Protein processing in endoplasmic reticulum and Neuroactive ligand-receptor interaction metabolic pathways (**Supplementary Figure S2A**). DEGs between the Ctrl and Alc groups showed significant enrichment in Cellular senescence, Oocyte meiosis, Cell cycle, and Tyrosine metabolism metabolic pathways (**Supplementary Figure S2B**). Furthermore, DEGs between the Perio and Perio+Alc groups were primarily associated with pathways involved in oxidative phosphorylation metabolism (**Supplementary Figure S2C**).

### 3.5 Chronic alcohol consumption reshapes the gingival metabolome and dysregulates amino acid, lipid, bile acid, and energy metabolism in periodontitis

**Figure 6:**
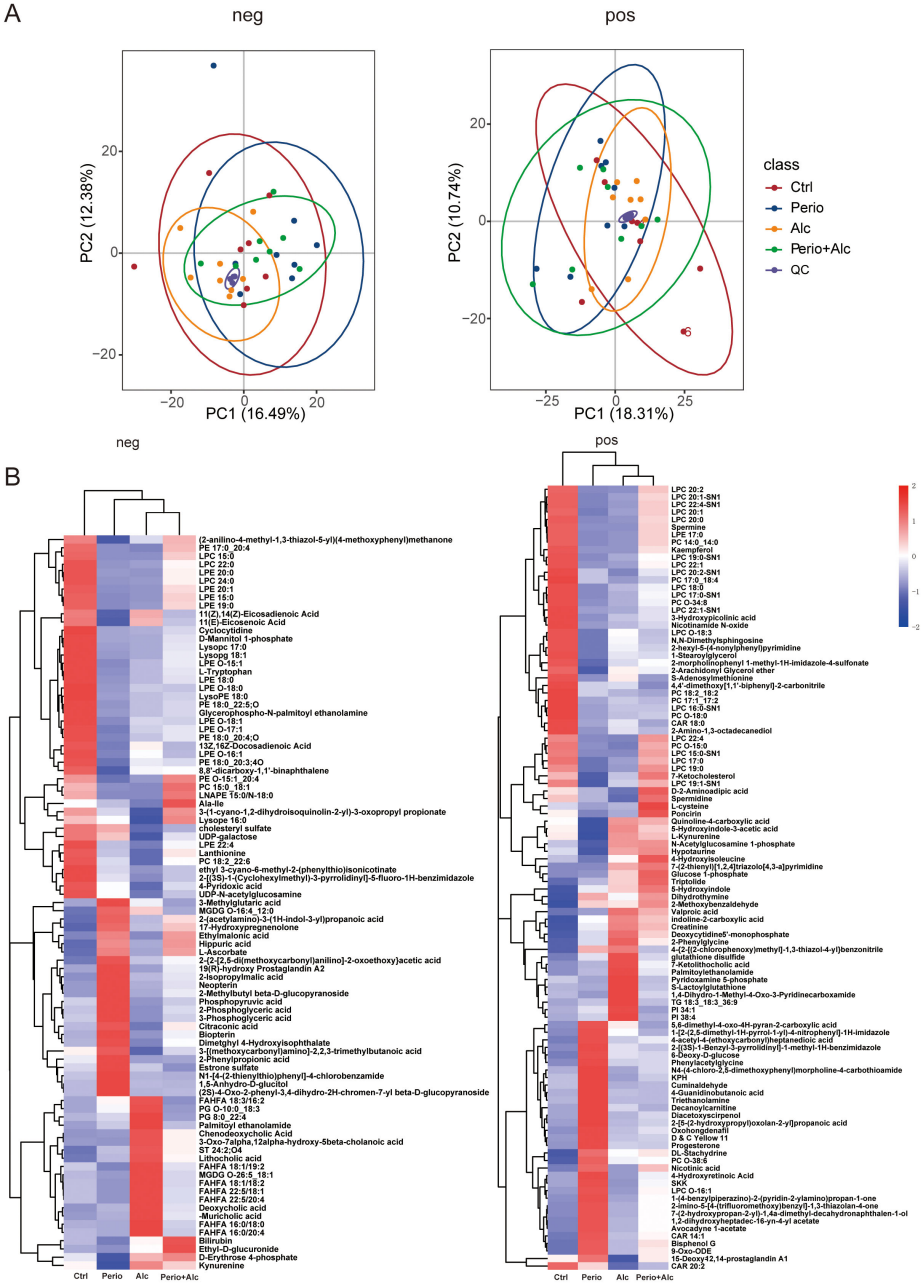
Chronic alcohol consumption disrupts the metabolic profile in rats with periodontitis. **(A)** Principal component analysis (PCA) of metabolomic profiles showing separation among the four experimental groups. PCA score plots derived from data acquired in negative ion mode (left) and positive ion mode (right). In both plots, colored symbols represent the four experimental groups, and purple symbols denote pooled quality control (QC) samples. The close clustering of QC samples indicates high analytical reproducibility, while the separation among experimental groups reflects treatment-related metabolic alterations. **(B)** Clustering heatmap of differential metabolites in neg and pos modes. Significantly altered metabolites are shown across all groups. Data from negative (left) and positive (right) ionization modes are displayed separately. Rows (metabolites) and columns (samples) are clustered to visualize abundance patterns and group similarities.

To elucidate metabolic alterations induced by chronic alcohol consumption during periodontitis progression, we conducted untargeted metabolomic profiling of rat gingival tissues using UPLC-MS. Analysis detected 611 metabolites in negative ion mode and 812 in positive ion mode. Principal component analysis (PCA) demonstrated robust analytical quality, evidenced by tight clustering of quality control (QC) samples, confirming data suitability for subsequent analyses. The PCA score plot revealed partial overlap among the groups (**Supplementary Figure S3**). Partial least squares-discriminant analysis (PLS-DA) further indicated separation of the Alc, Perio and Perio+Alc groups from controls (**Supplementary Figure S4**). Collectively, these multivariate analyses demonstrate distinct metabolite profiles across the experimental groups (**Figure 6A**).

Differential metabolites in rat gingival tissues across experimental groups were identified using a combined threshold of VIP score > 1, P-value < 0.05, and absolute fold change |log2FC| ≥ 1 (corresponding to FC ≥ 2 or FC ≤ 0.5). To visualize the overarching metabolic patterns across all four experimental groups, we generated a hierarchical clustering heatmap (**Figure 6B**). This analysis revealed 54 significant metabolites in negative ion mode for the Perio group versus control comparison (21 upregulated, 33 downregulated). In positive ion mode, 71 differential metabolites were identified (35 upregulated, 36 downregulated). Notably, several metabolites exhibited significant upregulation, including L-tryptophan, 4-guanidinobutyric acid, S-adenosylmethionine, spermidine, citric acid, L-ascorbic acid, novel purine derivatives, and progesterone. Conversely, urea was markedly downregulated (**Supplementary Figure S5**; **Supplementary Data Sheets S1, S2**).

Compared to the Ctrl group, the Alc group exhibited 41 differential metabolites in the negative ion mode, with 19 upregulated and 22 downregulated. In the positive ion mode, 125 differential metabolites were observed, with 56 upregulated and 69 downregulated. Significant upregulation was noted in metabolites such as ursodeoxycholic acid, deoxycholic acid, cholic acid, 7-ketocholic acid, hydroxy fatty acid esters, phosphatidylinositol, glutathione disulfide, and creatinine, whereas metabolites like lysophosphatidylcholine, phosphatidylcholine, uridine diphosphate galactose, UDP-N-acetylglucosamine, spermine, and spermidine were significantly downregulated (**Supplementary Figure S6**; **Supplementary Data Sheets S3, S4**).

A comparison between the Perio+Alc and Perio groups revealed 13 differentially abundant metabolites in negative ion mode, of which 4 were increased and 9 were decreased. In positive ion mode, 32 differential metabolites were identified, with 21 increased and 11 decreased. Collectively, chronic alcohol consumption altered metabolic pathways in the gingival tissues of rats with periodontitis. Key metabolites exhibiting significant increases included D-erythrose-4-phosphate, spermine, spermidine, 5-hydroxyindole-3-acetic acid, uric acid, bilirubin, and 7-ketocholesterol. Conversely, phosphoenolpyruvate, phenylacetic acid, and progesterone were significantly decreased (**Supplementary Figure S7**; **Supplementary Data Sheets S5, S6**).

Enrichment analysis of differential metabolites in metabolic pathways was conducted at a significance threshold of p<0.05. Differential metabolites identified across comparison groups were mapped to the KEGG database to elucidate their associated pathways. Annotated metabolites were then subjected to pathway enrichment analysis to identify pathways exhibiting significant metabolite accumulation. In the Perio group compared to the Ctrl group, differential metabolites predominantly annotated in the negative ion mode were significantly enriched in the 2-oxocarboxylic acid metabolism pathway, the biosynthesis pathways of valine, leucine, and isoleucine, and the choline metabolism in cancer pathway. In the positive ion mode, significant enrichment was observed for the choline metabolism and glycerophospholipid metabolism pathways (**Supplementary Figure S8**).

Comparisons between the Alc and Ctrl group revealed that differential metabolites detected in the negative ion mode were predominantly annotated and significantly enriched in the bile secretion pathway, as well as the amino sugar and nucleotide sugar metabolism pathways. In the positive ion mode, significant enrichment was observed for pathways including myo-inositol phosphate metabolism, the phosphatidylinositol signaling system, glutathione metabolism, tuberculosis-related pathways, glycerophospholipid metabolism, glycosylphosphatidylinositol anchor biosynthesis, and autophagy (**Supplementary Figure S9**).

**Figure 7:**
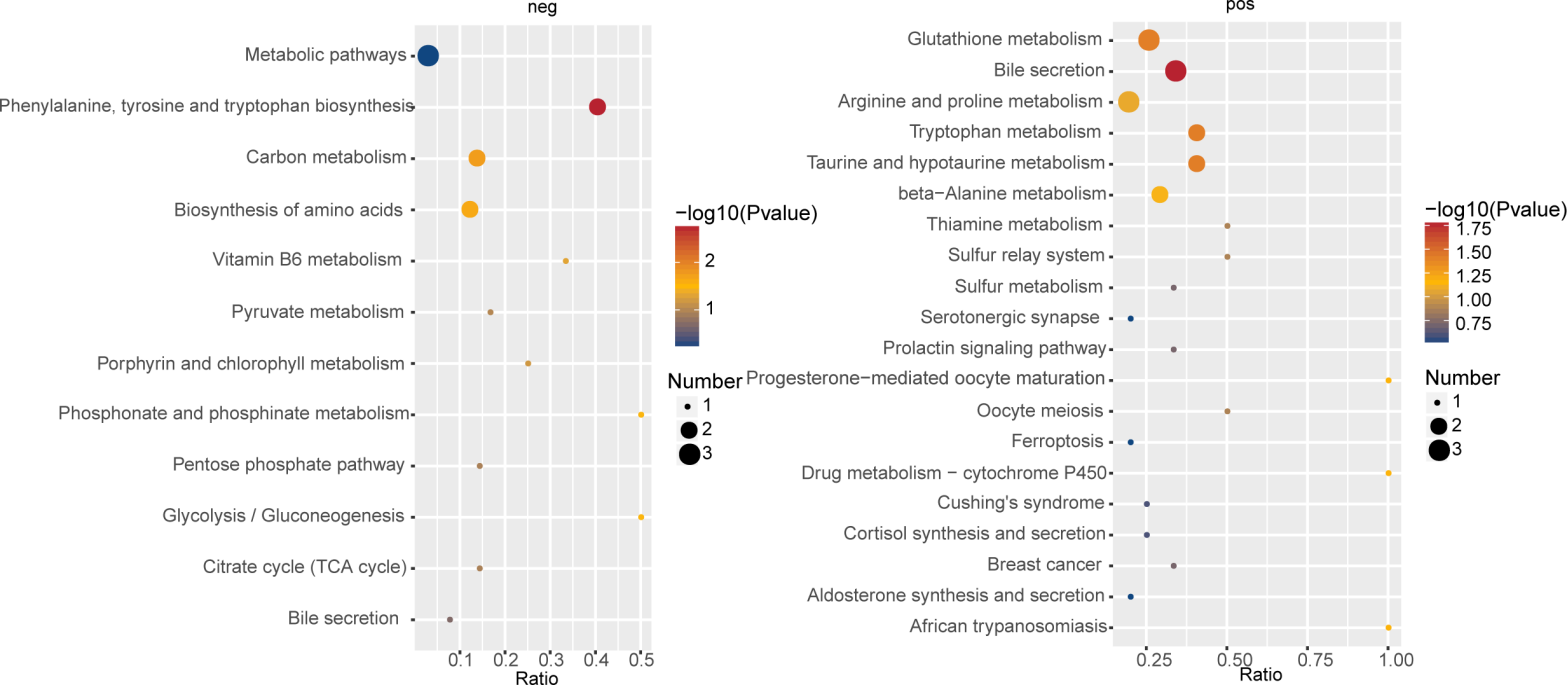
KEGG pathway enrichment analysis of differential metabolites between the Perio+Alc and Perio groups. The bubble plot displays KEGG pathways where the proportion of differential metabolites (x/n) exceeds the background proportion (y/N), a criterion adopted due to the limited metabolite set in untargeted analysis. The x-axis represents the enrichment factor (Rich Factor). The y-axis lists the enriched pathway terms. The size of each bubble corresponds to the number of metabolites mapped to that pathway. The color gradient indicates the statistical significance of enrichment, represented by -log_10_ (P value), with red denoting higher significance (P < 0.05, |FC|> 2).

Negative ion mode analysis revealed that differential metabolites between Perio+Alc and P groups were primarily mapped to phenylalanine, tyrosine, and tryptophan biosynthesis, alongside carbon metabolism, amino acid biosynthesis, glycolysis/gluconeogenesis, phosphonate and phosphinate metabolism, and vitamin B6 metabolism. Conversely, in the positive ion mode, enrichment was observed for bile secretion, tryptophan metabolism, taurine and hypotaurine metabolism, and glutathione metabolism (**Figure 7**).

### 3.6 Multi-omics integration reveals four core dysregulated pathways driving alcohol-exacerbated periodontitis

**Figure 8:**
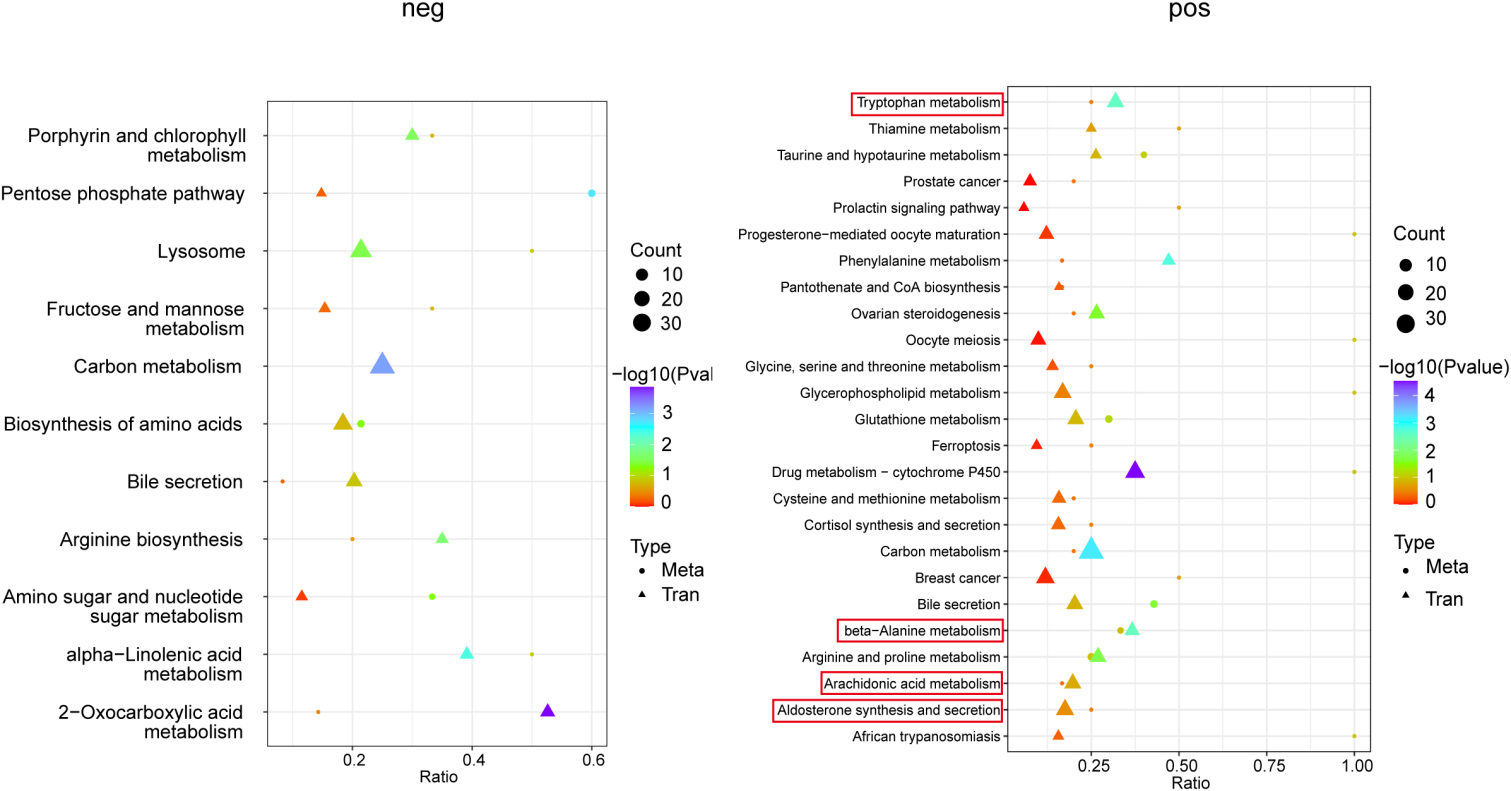
Integrated multi-omics pathway analysis reveals four core dysregulated pathways in the Perio+Alc vs. Perio comparison. The y-axis lists the enriched pathway terms. The x-axis represents the enrichment ratio. Dot size denotes the count of genes or metabolites enriched in each term; dot color reflects the statistical significance (−log_10_P); dot shape distinguishes the omics type (transcriptomics vs. metabolomics).

To investigate the impact of alcohol consumption on periodontal disease in rats, we mapped all identified differentially expressed genes and metabolites onto the KEGG pathway database. This analysis revealed shared pathway information, highlighting the key biochemical and signal transduction pathways implicated. Significant enrichment differences between the Perio group and Perio+Alc group were primarily observed in th beta- alanine metabolism, arachidonic acid metabolism, tryptophan metabolism, and aldosterone synthesis and secretion (**Figure 8**).

## 4 Discussion

This study integrates morphological, transcriptomic, and metabolomic analyses to evaluate periodontal tissue samples from rats subjected to chronic alcohol consumption, aiming to elucidate the pathophysiological mechanisms through which sustained ethanol intake exacerbates periodontal disease progression. Although specific metabolites and pathways linked to periodontitis have been identified in previous research, these studies predominantly utilized saliva or gingival crevicular fluid samples. Thus, research based on more tissue-derived samples, such as gingival tissue, remains limited.

Our findings demonstrate significantly elevated serum ALT and AST levels in the Alc and Perio+Alc groups compared to the Ctrl group. This elevation confirms successful modeling of chronic alcohol exposure and indicates substantial hepatic functional impairment. Under the present experimental conditions, periodontal inflammation exhibited a comparatively limited effect on liver function. The observed abnormalities in these hepatic injury biomarkers suggest that alcohol-induced systemic metabolic disturbances exacerbate local periodontal destruction (24). These results are consistent with the conclusions of de Almeida et al. (25) regarding alcohol-related hepatic injury, where prolonged ethanol intake activates osteoclasts, thereby accelerating alveolar bone loss in experimental periodontitis models. Consequently, when evaluating alcohol’s impact on periodontal tissues, its potential systemic effects on other organs must be considered. Supporting this notion, a population-based Korean nationwide cohort study reported elevated serum ALT and AST levels among both light-to-moderate drinkers and heavy drinkers (26).

Cross-sectional studies consistently report a positive correlation between alcohol consumption and periodontitis incidence, particularly severe forms, with drinkers demonstrating advanced disease severity (27, 28). A 2016 meta-analysis indicated that each additional gram of daily alcohol intake increases periodontitis risk by 0.4% (29). Recent data from the French NutriNet-Santé cohort further confirmed that alcohol consumers exhibit higher likelihood of severe periodontitis, correlating with increased spirits intake frequency and higher average daily ethanol consumption (30).

Histological analysis (HE staining) and micro-CT imaging revealed that chronic alcohol intake alone did not induce inflammation or alveolar bone resorption in healthy periodontal tissues. However, in rats with experimental periodontitis, chronic alcohol consumption exacerbated inflammatory responses and attachment loss. These findings suggest that alcohol-induced systemic inflammation and metabolic toxicity disrupt periodontal tissue repair mechanisms, accelerating disease progression, which is consistent with prior studies identifying alcohol as a key risk factor for periodontal deterioration (31). Ethanol exposure promotes bone resorption-formation imbalance, driving alveolar bone loss and dysregulated bone metabolism (31, 32), with some studies reporting bone resorption even without induced periodontitis (33). Conversely, low-concentration alcohol (5%) exerted negligible or potentially protective effects against alveolar bone loss in ligature-induced periodontitis models (34), possibly attributable to anti-inflammatory or bone-modulating properties (35).

To investigate the mechanisms by which chronic alcohol consumption exacerbates periodontitis, we performed RNA sequencing and untargeted metabolomics analyses on gingival tissues. Transcriptomic profiling was conducted under conditions of chronic alcohol intake and periodontal disease. Gene set enrichment analysis demonstrated that both chronic alcohol exposure and periodontitis independently induced significant differential gene expression in gingival tissues. Principal component analysis clearly distinguished groups, indicating that transcriptomic profiles were strongly associated with experimental conditions. GO enrichment analysis of DEGs highlighted biological processes relevant to alcohol consumption and periodontal pathology, while KEGG pathway analysis elucidated associated metabolic pathways.

Specifically, 39 DEGs between the Alc and Ctrl groups were significantly enriched in pathways related to spindle assembly, microtubule cytoskeleton organization, and mitotic cell cycle biological process. This suggests ethanol directly impairs gingival epithelial cell proliferation and barrier function, aligning with clinical observations of high oral mucosal lesion prevalence in chronic alcohol consumers (36). Mechanistically, the ethanol metabolite acetaldehyde induces abnormal tubulin acetylation (37), disrupting spindle formation and causing epithelial cell cycle arrest (38). Furthermore, García-Villaescusa et al. reported elevated salivary short-chain fatty acids (e.g., butyrate, isovalerate) in periodontitis patients, which inhibit gingival epithelial proliferation and delay barrier repair (39). Zhao et al. (40) observed increased abundance of pathogenic bacteria like Actinomyces in the oral cavity of alcohol-fed rats with periodontitis. These bacteria secrete cytoskeleton-disrupting toxins, potentially establishing a positive feedback loop with host cell cycle dysregulation. Supporting this, Joseph’s transcriptomic analysis (41) confirmed dysregulation of host cell cycle genes (e.g., CDK1, CCNB1) in periodontitis, increasing epithelial sensitivity to alcohol’s cytotoxic effects. Thus, the coexistence of chronic alcohol intake and periodontitis synergistically disrupts cell cycle regulation and amplifies microbial toxin effects, further impairing periodontal tissue regeneration.

These findings indicate that chronic alcohol consumption and periodontitis induce distinct gene expression patterns, with alcohol potentially modulating specific metabolic pathways that impact gingival tissue function and inflammatory responses. Between the Perio+Alc and Perio groups, 2,960 DEGs were significantly enriched in processes involving oxidation-reduction process, cellular respiration, oxidative phosphorylation, and ATP metabolic process biological processes. This enrichment is consistent with energy metabolism remodeling observed during periodontal disease progression. Mitochondrial dysfunction, leading to reduced ATP synthesis, may impair osteoblast activity, exacerbate tissue hypoxia, and promote alveolar bone resorption. Wang et al. (42) identified elevated salivary glucose and methylmalonic acid levels—markers of mitochondrial dysfunction—in late-stage periodontitis patients, reflecting impaired TCA cycle activity. Notably, the ethanol metabolite acetaldehyde directly inhibits complex I of the electron transport chain (43), resulting in excessive ROS production and establishing a detrimental cycle of oxidative stress and energy deficiency.

This study investigates the gingival metabolome in the context of chronic alcohol consumption and periodontitis, identifying potential metabolic biomarkers implicated in alcohol-aggravated experimental periodontitis. Our results demonstrate that numerous metabolites and specific metabolic pathways are modulated by either periodontitis or chronic alcohol exposure.

Non-targeted metabolomic profiling, employing both positive and negative ion modes for comprehensive metabolite detection (44), revealed a substantial number of significantly altered metabolites across the four groups (VIP > 1, P < 0.05, FC ≥ 2 or FC ≤ 0.5). Distinct metabolite expression patterns were observed under different ionization modes. Key metabolic disruptions differentiating the groups included dysregulated bile acid and lipid metabolism, amino acid metabolism imbalances, and energy metabolism anomalies.

Metabolomic analysis demonstrated significant accumulation of bile acids—including chenodeoxycholic acid (CDCA), deoxycholic acid (DCA), lithocholic acid (LCA), and 7-ketolithocholic acid (7-KLCA)—in the Alc group, with enrichment in the bile secretion pathway. This finding aligns with the emerging gut-liver axis concept in alcohol metabolism proposed by Professor Bin Gao’s team (24). While the liver remains the primary site of alcohol detoxification, approximately 30% of hepatic acetaldehyde (AcH) is excreted into the intestine via bile and metabolized by intestinal epithelial aldehyde dehydrogenase 2 (ALDH2). Chronic alcohol consumption disrupts this pathway, causing abnormal enterohepatic bile acid circulation.

Within the oral cavity, accumulated bile acids activate NLRP3 inflammasomes in gingival fibroblasts (45), promoting IL-1β release and amplifying periodontal inflammation. Notably, the Perio+Alc group exhibited significant upregulation of 7-ketolithocholic acid. This oxidized steroidal metabolite acts as an inflammatory mediator, promoting macrophage polarization toward the pro-inflammatory M1 phenotype and stimulating RANKL-dependent osteoclastogenesis (46).

Disrupted amino acid metabolism is a hallmark of alcohol-related periodontitis. Compared to the Perio group, the Perio+Alc group exhibited elevated concentrations of spermidine and spermine, but decreased levels of 2-phenylpropionic acid. Polyamines, comprising putrescine, cadaverine, spermidine, and spermine (47), are organic compounds implicated in the pathogenesis of various systemic diseases, such as periodontitis, chronic kidney disease, Alzheimer’s disease, and cancer (48). Notably, polyamine levels are elevated in periodontitis (49, 50), where they play a critical role in bacterial pathogenic mechanisms (51). Furthermore, the Perio+Alc group displayed increased levels of kynurenine, a key intermediate in the tryptophan degradation pathway (52). Kynurenine activates the aryl hydrocarbon receptor (AhR), thereby promoting osteoclast differentiation (53). Additionally, the increased degradation of host periodontal proteins observed in periodontitis may stem from upregulated activity of inflammatory proteases, such as matrix metalloproteinases (54). These enzymes break down periodontal proteins into peptides and amino acids, providing nutrients and energy for bacteria and ultimately exacerbating periodontal inflammation.

Metabolomic analysis revealed decreased concentrations of UDP-galactose and UDP-N-acetylglucosamine in the Alc group compared to the Ctrl group. These metabolites participate in glycosylation and energy metabolism (55). UDP-N-acetylglucosamine stimulates hyaluronic acid synthesis through hyaluronan synthase, thereby modulating the cellular microenvironment in pathologies such as vascular diseases and cancer (56). Relative to the Perio group, the Perio+Alc group exhibited significantly reduced phosphoenolpyruvate (PEP) levels alongside elevated D-erythrose 4-phosphate. Enzymes of the pentose phosphate pathway convert D-erythrose 4-phosphate into glyceraldehyde-3-phosphate and fructose-6-phosphate, supporting bacterial growth and enhancing pathogenicity (57). As a key glycolytic intermediate, PEP depletion indicates impaired glycolytic and tricarboxylic acid (TCA) cycle fluxes. These metabolic disruptions were corroborated by transcriptomic data revealing defective oxidative phosphorylation, collectively culminating in cellular energy deficits.

Integrative transcriptomic and metabolomic analyses revealed co-enrichment of differentially expressed genes and metabolites in pathways including beta- alanine metabolism, arachidonic acid metabolism, tryptophan metabolism, and aldosterone synthesis/secretion when comparing the Perio+Alc and Perio groups.

Within the β-alanine metabolism pathway, enrichment analysis identified two upregulated metabolites (spermidine and spermine) alongside one upregulated DEG (*Gad1*) and three downregulated DEGs (*Aldh3a1*, *Hibch*, *Cndp1*). Sustained accumulation of polyamines during chronic inflammation may disrupt tissue repair by modulating cellular migration and inflammatory responses, thereby promoting periodontal disease progression. For instance, spermidine regulates cell migration through polyamine metabolism genes like *AMD1 (*58). Elevated *GAD1* expression, which is known to promote invasiveness in oral squamous cell carcinoma (OSCC) through β-catenin nuclear translocation and MMP7 secretion (59), may similarly facilitate fibroblast migration and alveolar bone destruction in periodontitis. Downregulation of ALDH3A1 reduces antioxidant capacity, leading to aldehyde accumulation that activates the IL-6/STAT3 pathway and promotes epithelial-mesenchymal transition (EMT) and bone resorption (60), suggesting its utility as a periodontal disease progression biomarker. The enzyme encoded by *HIBCH* (3-hydroxyisobutyryl-CoA hydrolase) is essential for mitochondrial energy homeostasis; its downregulation may exacerbate periodontal energy deficits and inflammation, analogous to *HIBCH* mutation-induced Leigh syndrome (61). Additionally, diabetic patients carrying the *CNDP1* Mannheim shortest allelic variant exhibit reduced serum carnosinase, potentially intensifying microvascular damage in periodontal tissues (62).

Enrichment analysis of the arachidonic acid metabolism pathway revealed one upregulated metabolite [5-oxo-6,8,11,14-eicosatetraenoic acid (5-OxoETE)], one upregulated DEG (Pla2g4a), and two downregulated DEGs (Pla2g4e, Pla2g4f). As a key inflammatory mediator derived from the 5-lipoxygenase (5-LO) pathway, 5-Oxo-ETE exacerbates chronic inflammation by prolonging neutrophil infiltration (63). Neutrophils subsequently release proteases and reactive oxygen species (ROS), which directly damage periodontal tissues and drive alveolar bone loss and gingival recession (64). Upregulated Pla2g4a catalyzes arachidonic acid release, driving prostaglandin (PGE_2_) and leukotriene synthesis. This process activates the MAPK/ERK pathway, directly promoting periodontal bone destruction and inflammatory infiltration that exacerbate tissue degradation (65).Pla2g4e participates in membrane phospholipid remodeling and anti-inflammatory mediator synthesis, its downregulation likely impairs membrane repair and induces necroptosis (66). Its marked downregulation in aggressive periodontitis (AgP) patients may thus attenuate a crucial regulatory brake on inflammation (67). Similarly, Pla2g4f mediates phospholipid hydrolysis to release arachidonic acid, the precursor of inflammatory mediators. Significant downregulation of Pla2g4f in inflammatory diseases may reflect impaired lipid signaling, compromising epithelial barrier repair (68). This impairment may dysregulate cellular responses to inflammatory signals, thereby promoting M1 macrophage polarization and enhancing the secretion of pro-inflammatory cytokines, ultimately exacerbating periodontal bone destruction (69).

Enrichment analysis of the tryptophan metabolism pathway identified one upregulated metabolite [L-kynurenine (L-Kyn)], two upregulated DEGs (Ido1, DHTKD1), and three downregulated DEGs (Cat, HADHA, DLST). Targeted metabolomics indicates that dysregulated tryptophan-kynurenine metabolism in periodontitis patients modulates host-microbiome crosstalk and immune responses, thereby exacerbating inflammation (70). Notably, Ido1 expression is elevated in periodontal lesions versus healthy tissues. Induced by IFN-γ, IL-1β, TNF-α, and bacterial LPS, it degrades tryptophan to suppress T-cell activity and promote immune tolerance (71). DHTKD1 mutations impair mitochondrial function and correlate positively with inflammatory gene expression (72). The Cat gene encodes catalase, a key H_2_O_2_-scavenging enzyme. In diabetic periodontitis, its expression is further suppressed via Nrf2 pathway inhibition, accelerating tissue destruction. Pharmacological Nrf2 activation (e.g., by baicalein) upregulates catalase and reduces bone resorption (73). HADHA-mediated fatty acid β-oxidation is essential for mitochondrial ATP synthesis; its downregulation causes ROS accumulation, energy deficit, and apoptosis (74). Similarly, DLST, a pyruvate dehydrogenase complex component, is significantly downregulated in periodontitis. This deficiency disrupts glycolysis-TCA cycle coupling, exacerbating energy crises and oxidative stress (75).

Enrichment analysis of the aldosterone synthesis and secretion pathway revealed one downregulated metabolite [progesterone (PG)], one upregulated DEG (Atf6b), and two downregulated DEGs (Lipe, Adcy2). Alcohol intake (0.5 g/kg) reduces progesterone levels (76), potentially through elevated hepatic NAD^+^/NADH ratios that inhibit steroidogenesis (77). Progesterone exerts a protective effect on alveolar bone by modulating the RANKL/OPG system to suppress osteoclastogenesis. Consequently, reduced progesterone levels compromise this regulatory mechanism, leading to increased osteoclast activity and accelerated bone resorption (78). *Atf6b*, a key regulator of endoplasmic reticulum stress and the unfolded protein response (UPR), is upregulated in periodontal inflammation models. Overexpression of Atf6b promotes the release of inflammatory cytokines, including IL-6 and TNF-α, and drives osteoclast differentiation and bone resorption (79). Lipe encodes lysosomal acid lipase (LAL). Deficiency in this enzyme impairs tissue repair and accelerates periodontal destruction (80). Adcy2, which mediates cAMP production, shows significantly reduced expression in inflammatory conditions. Diminished Adcy2 activity likely weakens anti-inflammatory signaling, facilitating periodontal disease progression (81).

This study identifies novel therapeutic targets for alcohol-exacerbated periodontitis; however, certain limitations persist. Animal models cannot fully replicate human alcohol consumption patterns, and clinical drinking behaviors exhibit significant heterogeneity. Furthermore, the causal relationships between key pathways, including tryptophan metabolism and osteoclastic activity, require further experimental validation within periodontal cell models. The diagnostic utility of biomarkers, such as spermidine, spermine, 5-oxo-ETE, L-citrulline, and progesterone, should be confirmed through population-based cohort studies.

In addition, as this study employed an untargeted metabolomics approach, limitations in metabolite identification should be acknowledged. Data were processed under stringent quality control criteria, and metabolite annotation followed the Metabolomics Standards Initiative guidelines. Compounds supported by MS/MS spectral matching were classified as Level 2, whereas those based solely on accurate mass were assigned as Level 3; no metabolites were validated with authentic standards (Level 1). Therefore, despite strict matching thresholds, potential misannotation—particularly among isomeric or structurally similar compounds—cannot be fully excluded and may influence the accuracy of biological interpretation. Future studies incorporating targeted metabolomics and validation with authentic standards are necessary to improve the reliability and reproducibility of these findings.

This work aims to facilitate the development of more targeted and effective strategies for preventing and managing periodontitis.

## 5 Conclusion

In summary, this study combines transcriptomic and metabolomic approaches to elucidate the complex pathogenic mechanisms through which chronic alcohol consumption exacerbates periodontitis in rats. The findings reveal novel insights into molecular pathways underlying alcohol-aggravated periodontal deterioration and lay the groundwork for identifying potential therapeutic targets. Future research should prioritize validating key regulatory pathways both *in vivo* and *in vitro*, as well as investigating their synergistic effects with established periodontal treatments such as subgingival debridement, thereby advancing clinical management strategies for this disease.

## Data Availability

The datasets presented in this study can be found in online repositories. The names of the repository/repositories and accession number(s) can be found below: PRJNA1328937 (SRA) and OMIX011943 (OMIX).

## Supplementary material

Supplementary file 1 Metabolomic analysis.
