# Supplementary material for: Integrated transcriptomics and metabolomics reveal the effects of chronic alcohol consumption on periodontitis in rats
Source: Front Immunol. 2026 May 18;17:1735355. doi: 10.3389/fimmu.2026.1735355 (PMC13223609; doi:10.3389/fimmu.2026.1735355)
Supplement: Supplementary file 6 [file Table1.docx]

**Integrated transcriptomics and metabolomics reveal the effects of chronic alcohol consumption on periodontitis in rats**

**Zirui Zhao****, Zixuan Zhao, Yaqian Zhai, Jingyuan Sun, Na Liu*, Qing Liu***

Hebei Key Laboratory of Stomatology/ Hebei Technology Innovation Center of Oral Health, School and Hospital of Stomatology, Hebei Medical University, Shijiazhuang, Hebei, China

*** Correspondence:**Qing Liu

liuqing@hebmu.edu.cn

Na Liu

liuna@hebmu.edu.cn

**Methods**

LC-MS Metabolomic Analysis of Gingival Samples

**Results**

Differentially Abundant Metabolites

**Figure legends**

**Fig.S1** Effects of alcohol consumption and periodontitis on the gingival transcriptome between Perio vs. Ctrl and Alc vs. Ctrl comparisons.

**Fig.S2** KEGG pathway enrichment analysis of differentially expressed genes.

**Fig.S3** PCA-Based Comparative Analysis of Metabolomic Signatures Across Groups.

**Fig.S4** Pairwise group comparisons of metabolomic data visualized by Partial Least Squares Discriminant Analysis (PLS-DA).

**Fig.S5** Clustering heatmap of differential metabolites between the Perio and the Ctrl groups.

**Fig.S6** Clustering heatmap of differential metabolites between the Alc and the Ctrl groups.

**Fig.S7** Clustering heatmap of differential metabolites between the Perio+Alc and the Perio groups.

**Fig.S8** KEGG pathway enrichment analysis of differential metabolites between the Perio and Ctrl groups.

**Fig.S9** KEGG pathway enrichment analysis of differential metabolites between the Alc and Ctrl groups.

**LC-MS Metabolomic Analysis of Gingival Samples**

**Extraction of Metabolites**

Gingival tissue samples were retrieved from storage at -80°C and thawed on ice. A 100 mg aliquot was weighed, pulverized in liquid nitrogen, and homogenized in 500 μL of 80% (v/v) aqueous methanol. The mixture was vortexed vigorously and incubated on ice for 5 minutes. Following incubation, the sample was centrifuged at 15,000 *g* and 4°C for 20 minutes. An aliquot of the resulting supernatant was diluted with mass spectrometry-grade water (MS-grade water) to adjust the methanol concentration to 53% (v/v). This diluted solution was centrifuged again under identical conditions (15,000 *g*, 4°C, 20 min). The final supernatant was collected for subsequent analysis (e.g., by LC-MS/MS).

**Preparation of Quality Control and Blank Samples**

Equal aliquots from sample were pooled to generate four quality control (QC) samples. For blank samples, periodontal tissue was substituted with 53% (v/v) aqueous methanol solution, which was then processed following identical procedures as described in the previous section.

**Ultra-high-performance liquid chromatography-tandem mass spectrometry (UHPLC-MS/MS) Analysis**

Chromatographic separation was performed on a Hypersil Gold C18 column (reversed-phase) maintained at 40°C, using a flow rate of 0.2 mL/min. For positive ion mode analysis, mobile phase A consisted of 0.1% (v/v) formic acid in water and mobile phase B was methanol. For negative ion mode analysis, mobile phase A was 5 mM ammonium acetate (pH 9.0) in water and mobile phase B was methanol. The gradient elution program was as follows: 0–1.5 min, 98% A / 2% B; 1.5–3 min, linear gradient to 15% A / 85% B; 3–10 min, linear gradient to 0% A / 100% B; 10–10.1 min, linear gradient to 98% A / 2% B; 10.1–12 min, hold at 98% A / 2% B.

Analytes eluting from the chromatographic column were detected using tandem mass spectrometry with a scan range of *m/z* 100–1500. Key parameters included an electrospray ionization (ESI) voltage of 3.5 kV, a capillary temperature of 320 °C, and an auxiliary gas temperature of 350 °C. To assess system stability throughout the analytical sequence, one quality control (QC) sample was analyzed after every eight experimental samples.

**Data preprocessing, compound Identification, and confidence assessment**

Raw mass spectrometry data files (.raw format) were imported into Compound Discoverer 3.3 software (Thermo Fisher Scientific) for processing. A series of filtering criteria were applied to each metabolite feature, including retention time and *m/z* ratio. The first quality control (QC) sample served as the reference for peak area calibration. Stringent quality thresholds were enforced, including a mass accuracy tolerance of 5 ppm, a signal intensity deviation limit of < 30% across QC samples, minimum signal intensity thresholds, and consideration of specific adduct ions. Background ions detected in blank samples were subtracted. Raw quantitative data were normalized to calculate relative peak areas. Metabolites exhibiting a coefficient of variation (CV) > 30% across QC samples were excluded, yielding the final dataset for metabolite identification and relative quantification.

Metabolite identification was performed using a tiered approach according to the Metabolomics Standards Initiative (MSI) guidelines. Target ions were integrated, and preliminary molecular formulas were predicted based on molecular ion peaks and fragmentation patterns. Experimental data were subsequently matched against online databases, including mzCloud, mzVault, and Masslist. Compounds achieving MS/MS spectral match scores > 70% with mass errors < 5 ppm against reference libraries (mzCloud and mzVault) were annotated as Level 2 (putatively annotated compounds). For features lacking confident MS/MS matches, accurate mass searches (mass error < 5 ppm) were conducted against the Masslist database, with matched entries assigned as Level 3 (Putatively Characterized Compound Classes). Unmatched features or those failing quality criteria were excluded from further analysis.

**Data statistical analysis** **and Bioannotation**

Identified metabolites were bioannotated and subjected to metabolic pathway analysis by querying established databases, including the Kyoto Encyclopedia of Genes and Genomes (KEGG; https://www.genome.jp/kegg/pathway.html), Human Metabolome Database (HMDB; https://hmdb.ca/metabolites), and LIPID MAPS Lipidomics Gateway (http://www.lipidmaps.org).

Metabolomics data were processed using metaX software. Subsequent multivariate and univariate statistical analyses were performed in R software (version 3.4.3). Multivariate analyses included principal component analysis (PCA) and partial least squares-discriminant analysis (PLS-DA). Variable importance in projection (VIP) scores were calculated for each metabolite. Univariate analysis involved Student's t-tests to determine P-values and fold changes (FC) between groups. Differential metabolites were selected based on the criteria: VIP > 1, P -value < 0.05, and |log₂(FC)| ≥ 1 (equivalent to FC ≥ 2 or FC ≤ 0.5).

Metabolite data were normalized via z-score transformation. Hierarchical clustering was then performed, and results were visualized using heatmaps. Functional annotation and pathway enrichment analysis of differential metabolites were conducted using the KEGG database. For KEGG enrichment analyses, only terms containing at least 3 metabolites were retained, with a significance threshold of P < 0.05.

**Fig.S1**


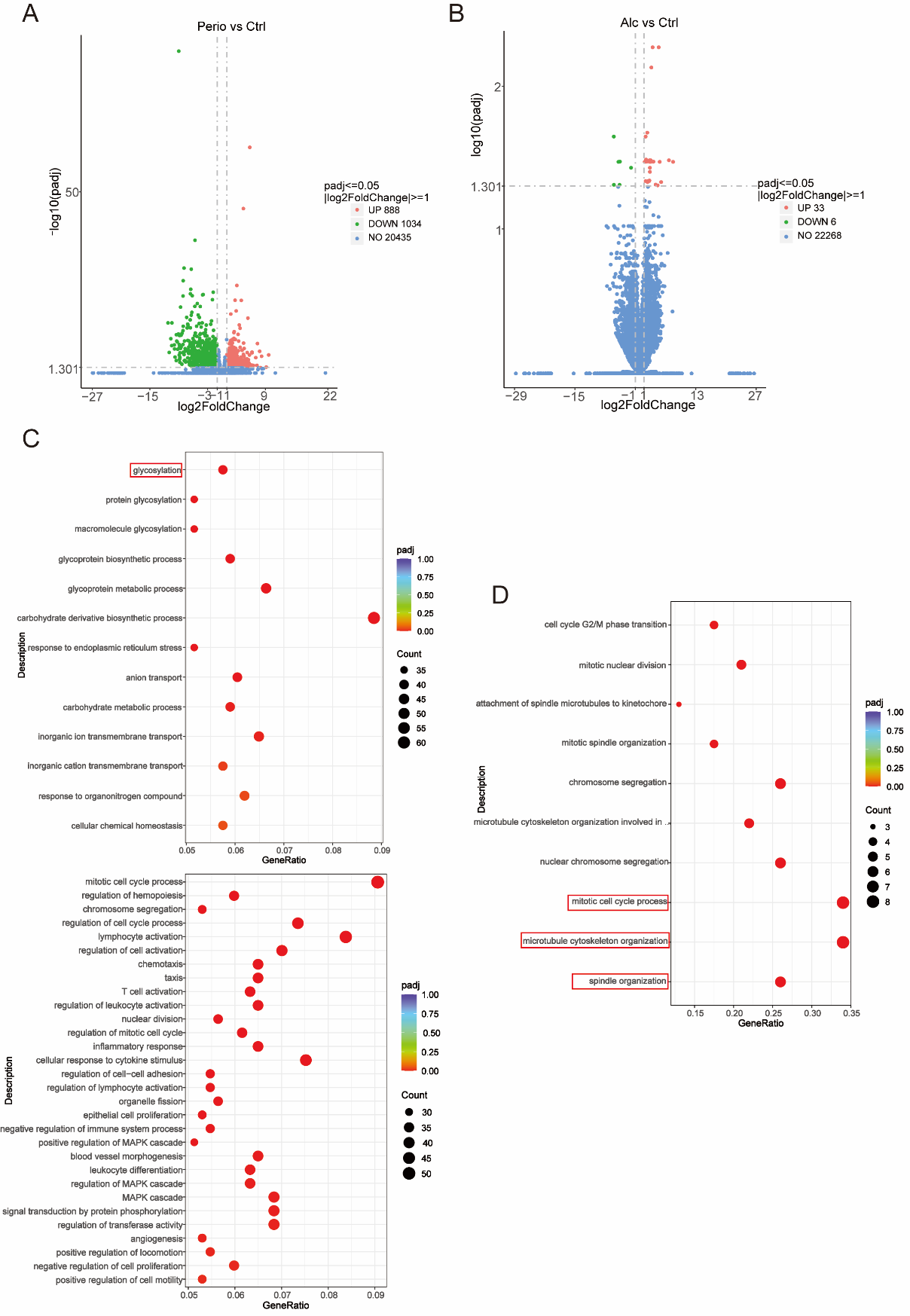


**Fig.S1** Effects of alcohol consumption and periodontitis on the gingival transcriptome between Perio vs. Ctrl and Alc vs. Ctrl comparisons. **(A)** Results of the differential expression analysis between the Perio and Ctrl groups are visualized in the volcano plot, which identifies 1,922 DEGs (888 upregulated; 1,034 downregulated). **(B)** The volcano plot depicts the minimal transcriptomic response in the Alc vs. Ctrl comparison, with only 39 DEGs (33 upregulated, 6 downregulated). **(C)** Gene Ontology (GO) enrichment analysis of downregulated (top) and upregulated (bottom) differentially expressed genes in the Perio vs. Ctrl comparison (P < 0.05, GeneRatio > 0.05, |FC|> 2). **(D)** Gene Ontology (GO) enrichment analysis of differentially expressed genes in the Alc vs. Ctrl comparison (P < 0.05, GeneRatio > 0.05, |FC|> 2).

**Fig.S2
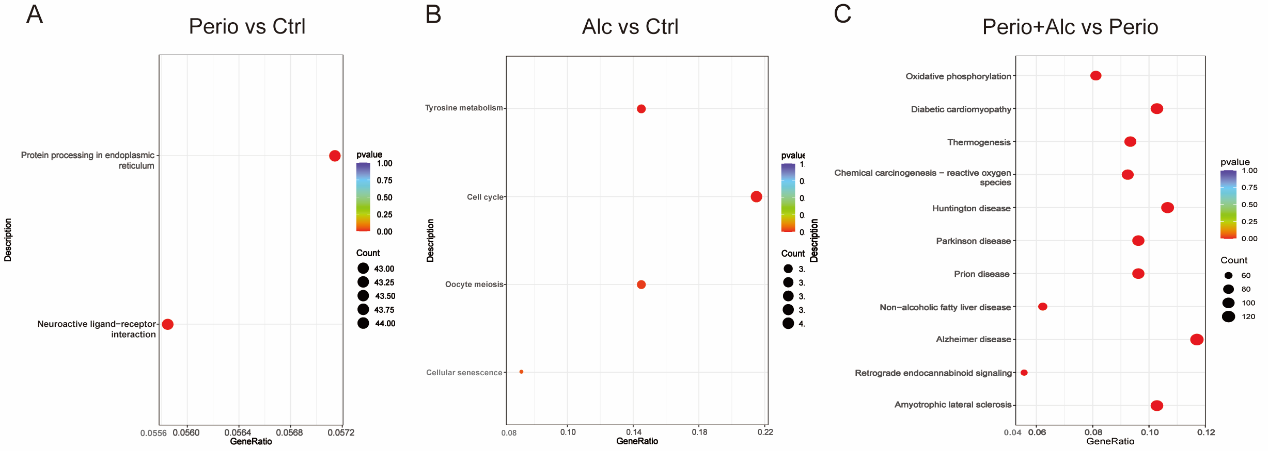
**

**Fig.S2** KEGG pathway enrichment analysis of differentially expressed genes. Scatter plot displays the top 20 most significantly enriched KEGG pathways (adjusted P < 0.05) for three key comparisons: **(A)** Perio vs. Ctrl, **(B)** Alc vs. Ctrl, and **(C)** Perio+Alc vs. Perio. The x-axis represents the Gene Ratio (number of differentially expressed genes mapped to a pathway divided by the total number of input genes). The y-axis lists the enriched KEGG pathway terms. The size of each bubble corresponds to the number of genes enriched in that pathway. The color gradient indicates the statistical significance of enrichment (adjusted P value), with red representing the most significant (P < 0.05, GeneRatio > 0.05, |FC|> 2).

**Fig.S3**
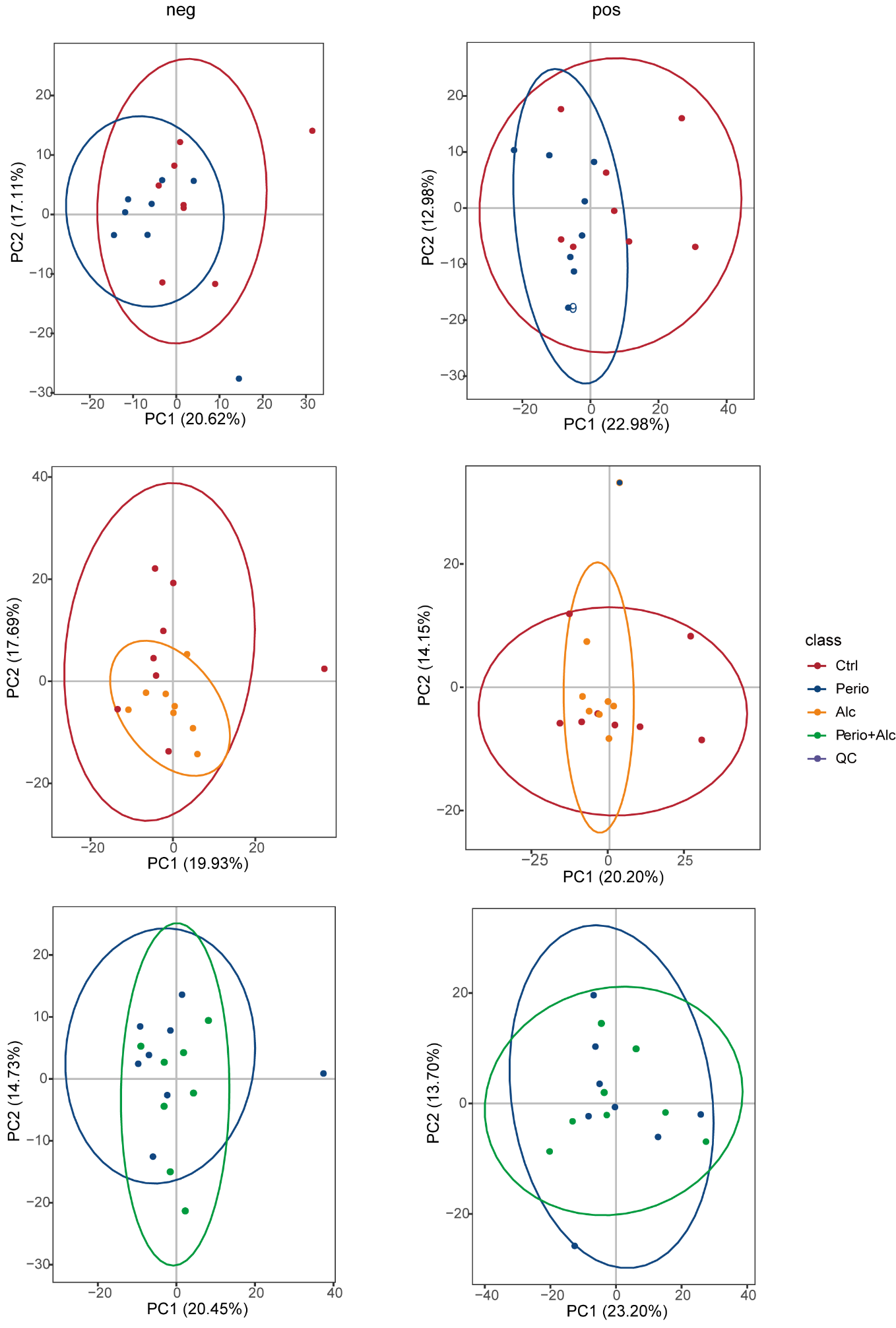


**Fig.S3** PCA-Based Comparative Analysis of Metabolomic Signatures Across Groups. PCA score plots illustrate the separation between specific experimental groups. Data from negative (neg, left panels) and positive (pos, right panels) ionization modes are shown separately. Each plot demonstrates the metabolic distinction between two groups under comparison.

**Fig.S4**


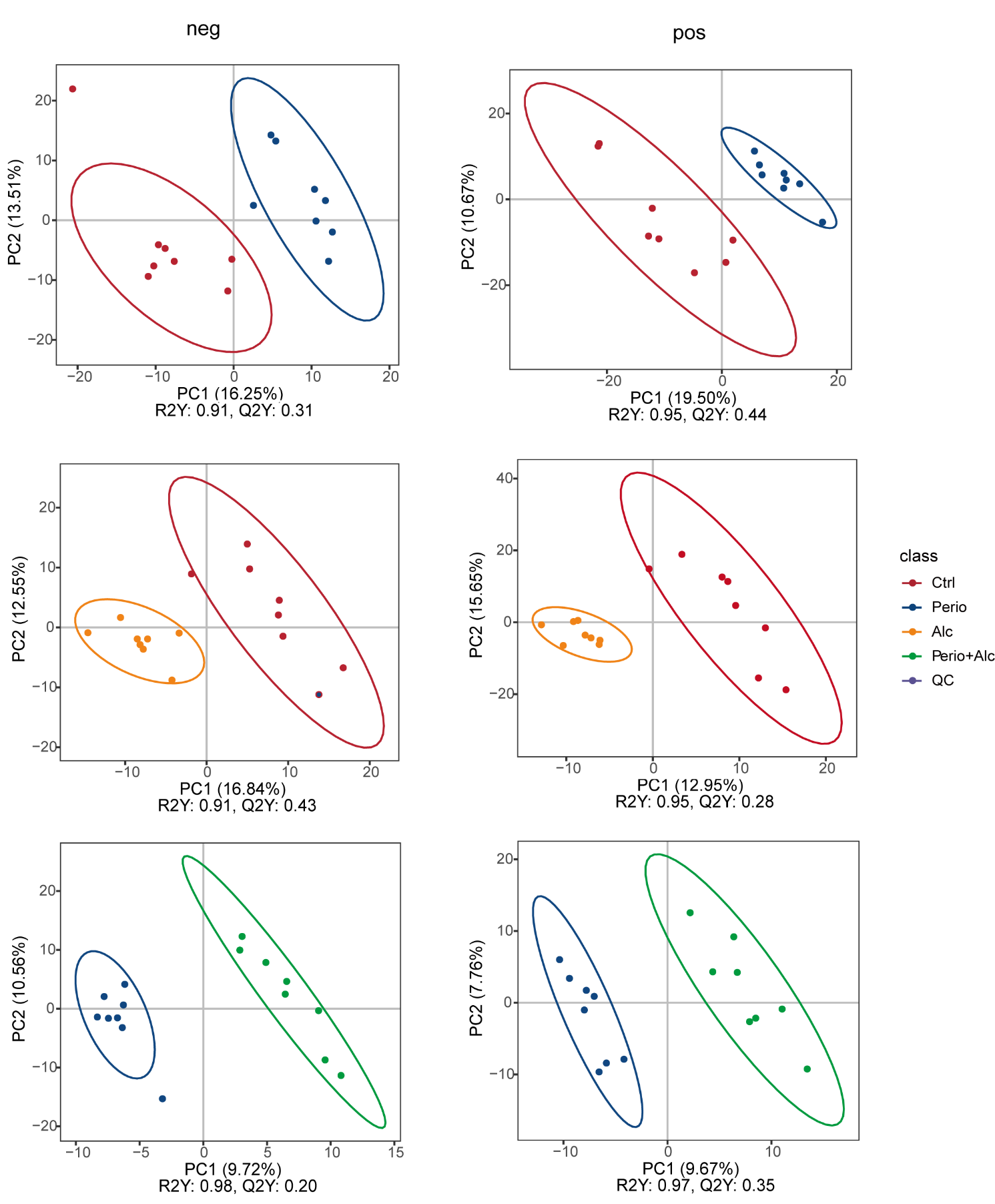


**Fig.S4** Pairwise group comparisons of metabolomic data visualized by Partial Least Squares Discriminant Analysis (PLS-DA). PLS-DA score plots for key pairwise comparisons are shown. The left and right columns present data from negative (neg) and positive (pos) ionization modes, respectively. As a supervised method, PLS-DA models were constructed to maximize the metabolic separation between the two specified groups in each plot, highlighting the most relevant variables for discrimination.

**Fig.S5**


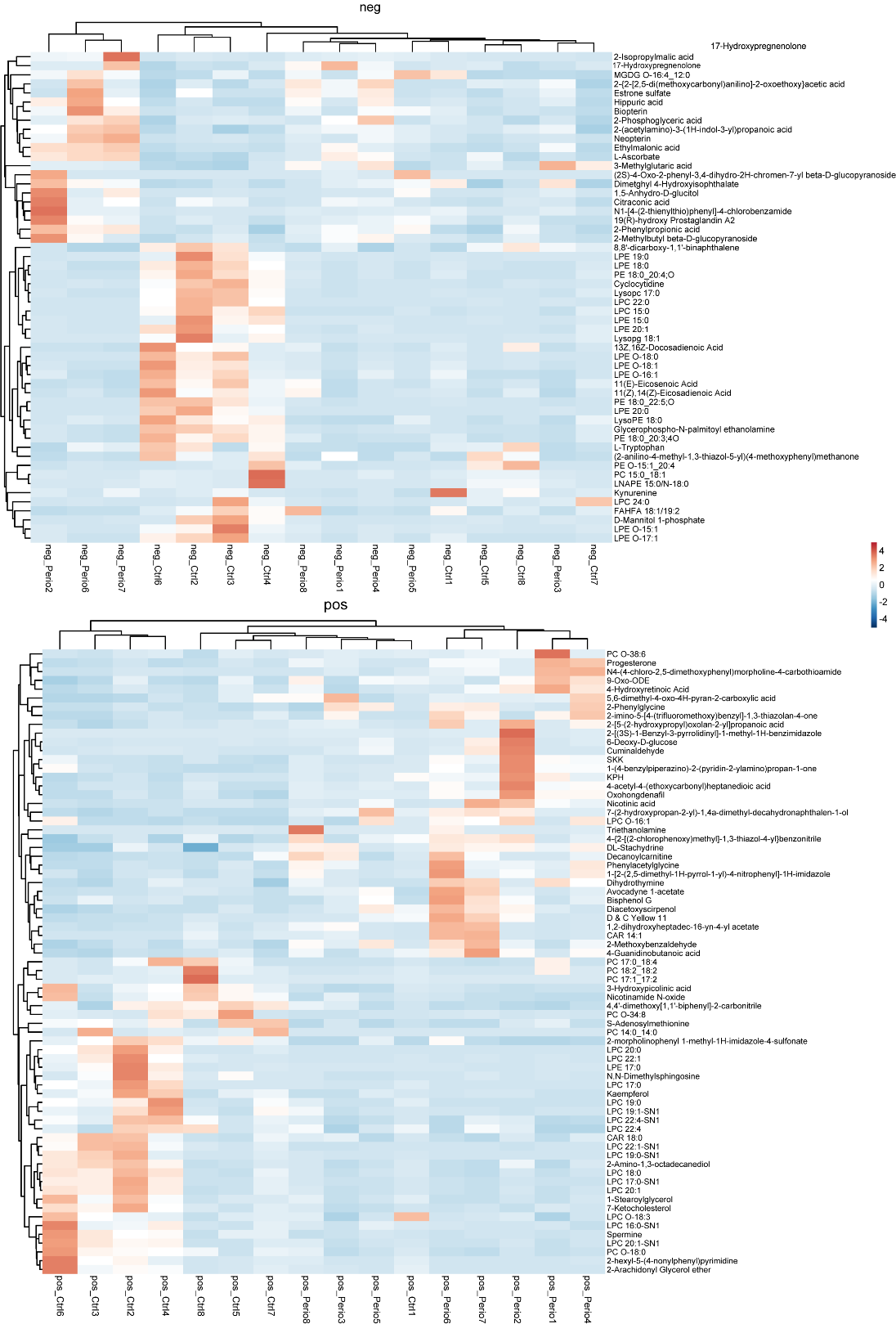


**Fig.S5** Clustering heatmap of differential metabolites between the Perio and the Ctrl groups. The heatmap displays the relative abundance (Z-score) of metabolites that were significantly altered in the Perio group compared to the Ctrl group. Data acquired in negative (top) and positive (bottom) ionization modes are shown separately. Each row represents a metabolite, and each column represents an individual biological sample. The color gradient from blue to red indicates low to high relative abundance.

**Fig.S6
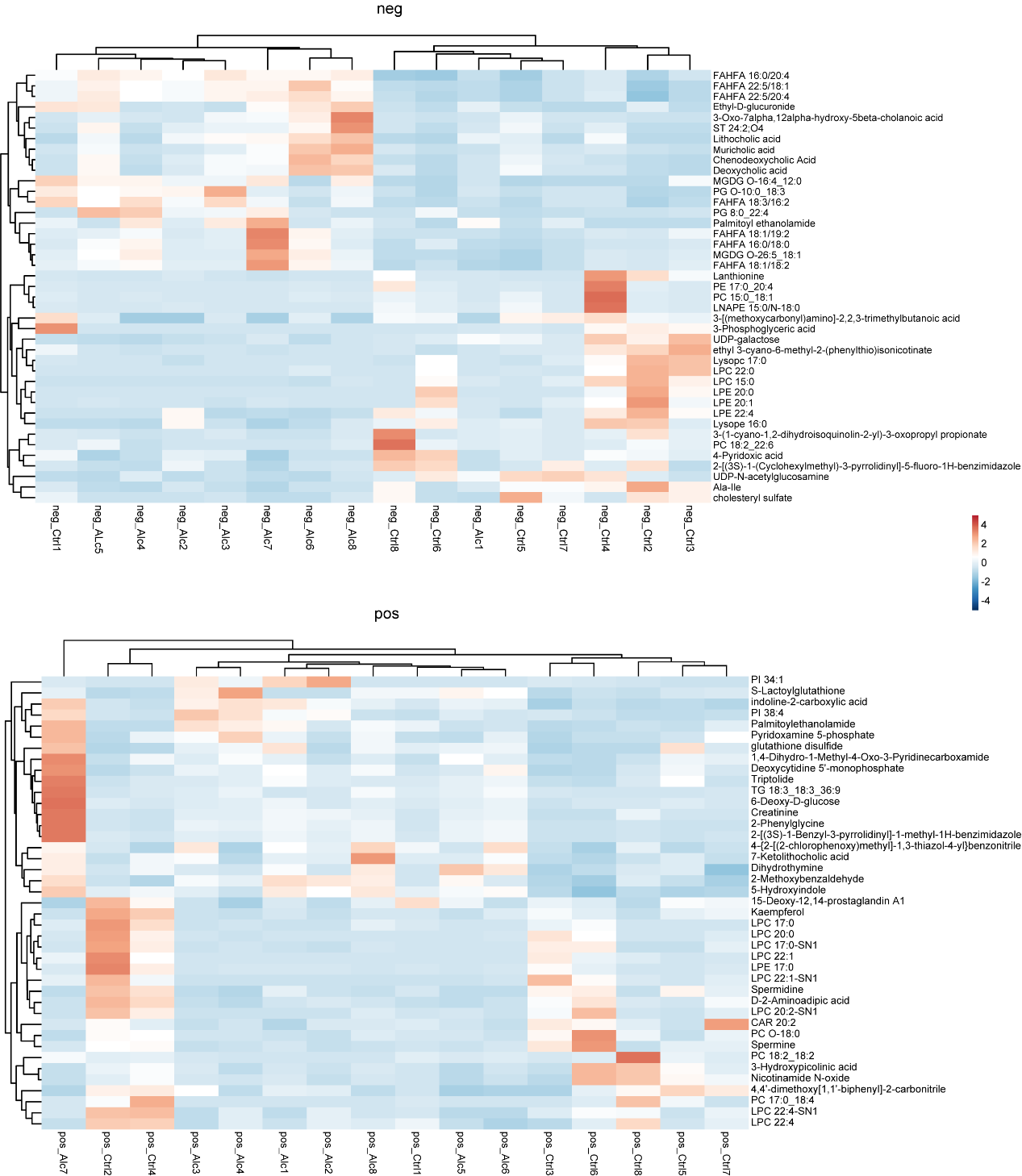
**

**Fig.S6** Clustering heatmap of differential metabolites between the Alc and the Ctrl groups. Shown are significantly altered metabolites from the Alc vs. Ctrl comparison, presented separately for neg (top) and pos (bottom) ionization modes. Rows (metabolites) and columns (samples) are clustered. The distinct grouping of samples visually underscores the metabolic divergence induced by alcohol intake.

**Fig.S7**
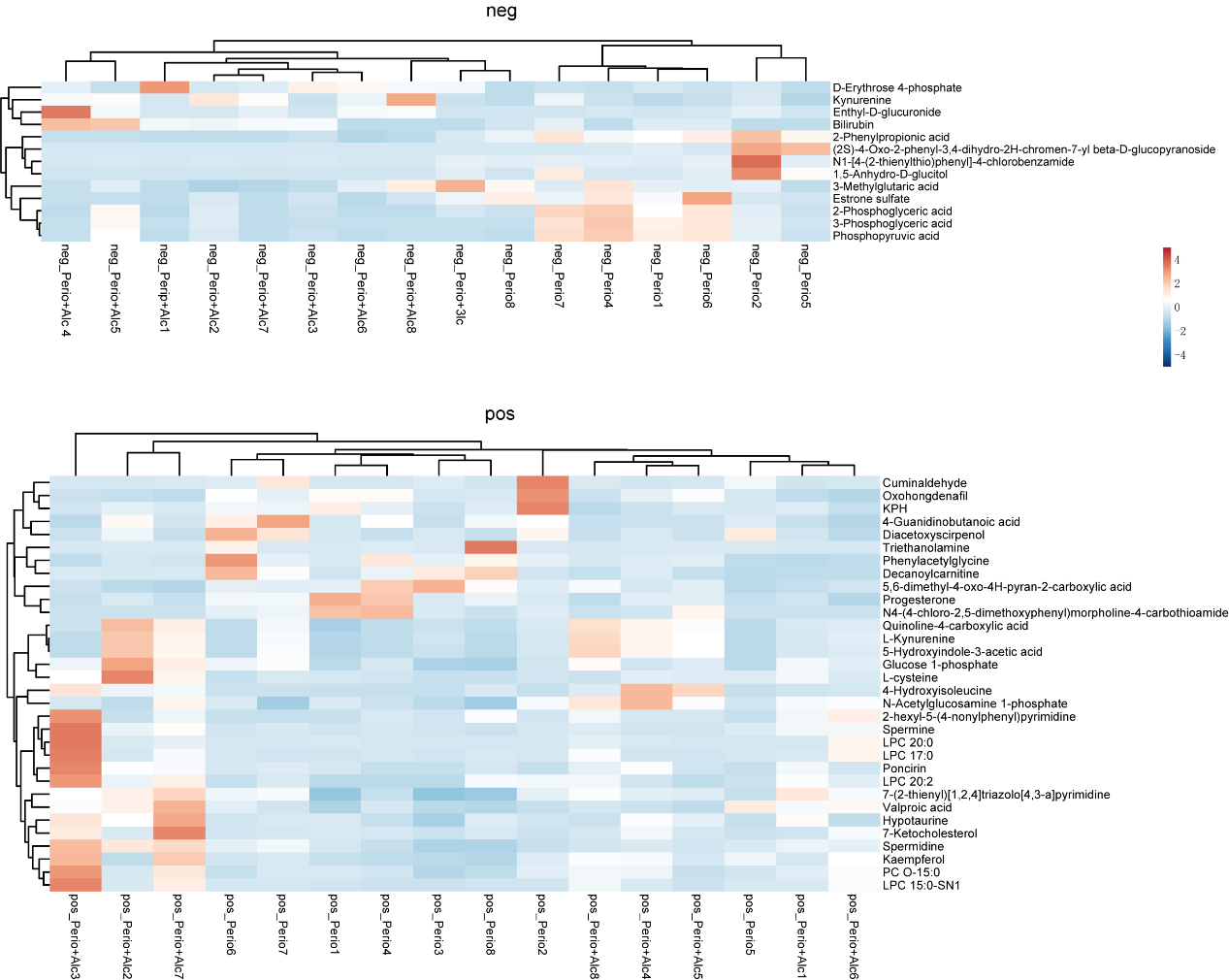


**Fig.S7** Clustering heatmap of differential metabolites between the Perio+Alc and the Perio groups. Metabolites differentially abundant due to the addition of alcohol to periodontitis are shown for neg (top) and pos (bottom) modes.

**Fig.S8**

**
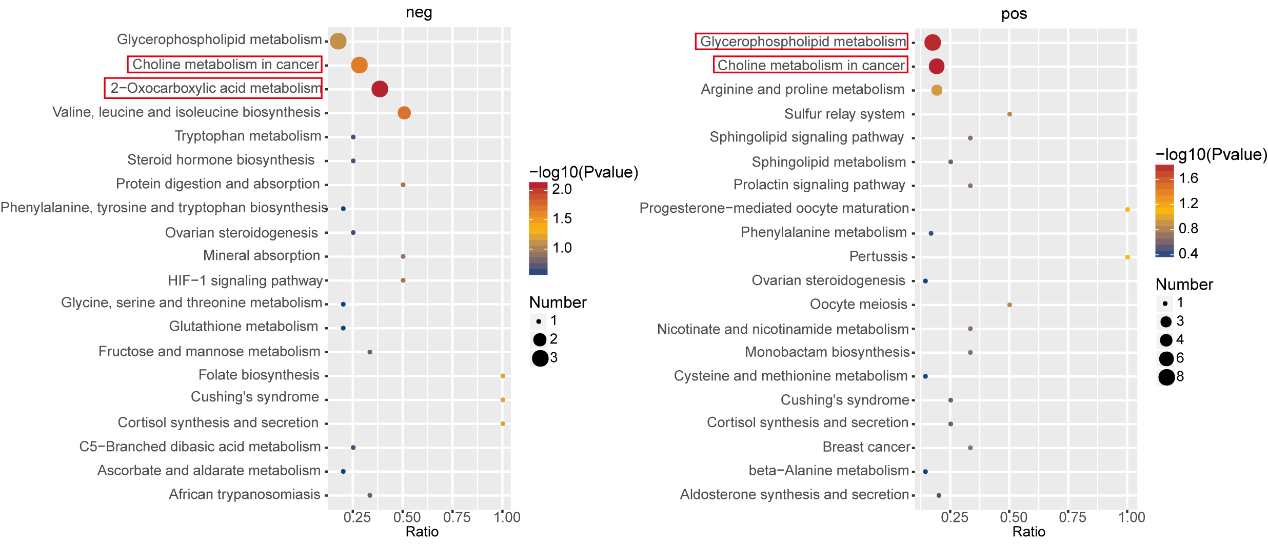
Fig.S8** KEGG pathway enrichment analysis of differential metabolites between the Perio and Ctrl groups. The bubble plot displays KEGG pathways that were considered enriched under a proportional criterion(x/n>y/N), which was applied due to the limited metabolite set in this untargeted analysis. Here, ‘N ’denotes the total number of metabolites with KEGG annotations, ‘n’ is the number of differential metabolites among ‘N’, ‘y’ is the number of metabolites annotated to a given pathway, and ‘x’ is the number of differential metabolites enriched in that pathway. The x‑axis represents the enrichment factor (Rich Factor). The y‑axis lists the enriched pathway terms. The size of each bubble corresponds to the number of metabolites mapped to the pathway, and the color gradient indicates the degree of enrichment represented by ‑log₁₀(P value), with red denoting higher significance.

**Fig.S9**

**
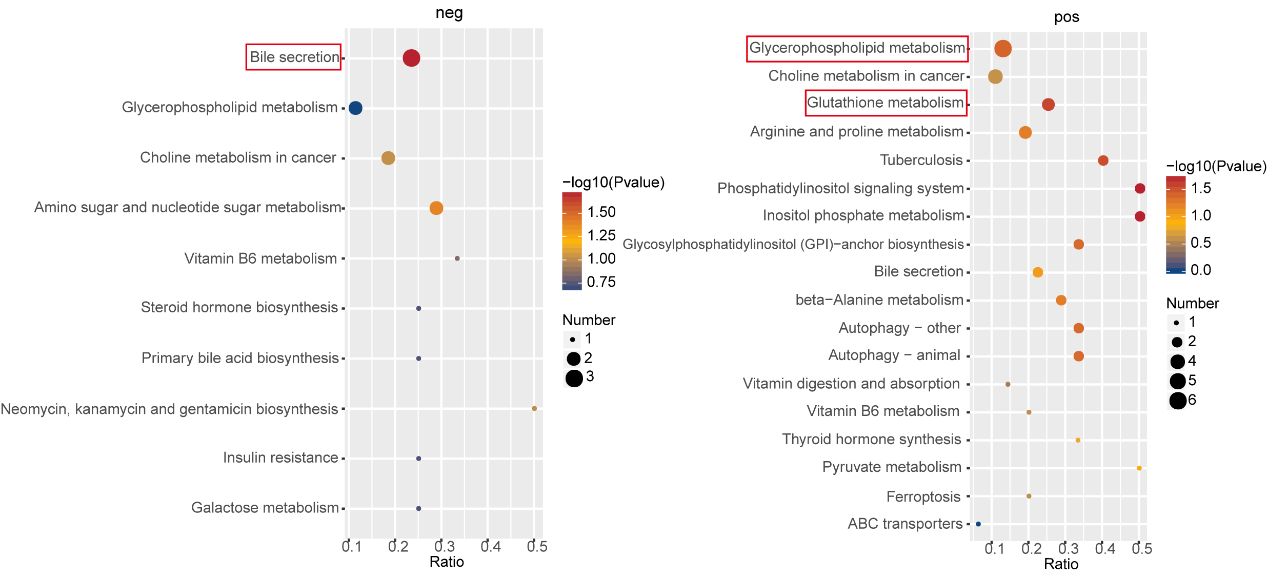
Fig.S9** KEGG pathway enrichment analysis of differential metabolites between the Alc and Ctrl groups. The bubble plot displays KEGG pathways where the proportion of differential metabolites (x/n) exceeds the background proportion (y/N), a criterion adopted due to the limited metabolite set in untargeted analysis. The x-axis represents the enrichment factor (Rich Factor). The y-axis lists the enriched pathway terms. The size of each bubble corresponds to the number of metabolites mapped to that pathway. The color gradient indicates the statistical significance of enrichment, represented by -log₁₀ (P value), with red denoting higher significance.
